# Potentiation of Antibiotics by a Novel Antimicrobial Peptide against Shiga Toxin Producing *E. coli* O157:H7

**DOI:** 10.1038/s41598-020-66571-z

**Published:** 2020-06-22

**Authors:** Juan Puño-Sarmiento, Erin M. Anderson, Amber J. Park, Cezar M. Khursigara, Debora E. Barnett Foster

**Affiliations:** 1 Department of Chemistry and Biology, Ryerson University, Toronto, Ontario Canada; 2 Department of Microbiology, Universidade Estadual de Londrina, Londrina, Paraná Brazil; 3 Department of Molecular and Cellular Biology, University of Guelph, Guelph, Ontario Canada; 4 Molecular and Cellular Imaging Facility, University of Guelph, Guelph, Ontario Canada; 5 Oral Microbiology, Faculty of Dentistry, University of Toronto, Toronto, Ontario Canada

**Keywords:** Antimicrobials, Gastroenterology

## Abstract

Infection with Shiga toxin-producing *Escherichia coli* (STEC) results in hemorrhagic colitis and can lead to life-threatening sequelae including hemolytic uremic syndrome (HUS). Conventional treatment is intravenous fluid volume expansion. Antibiotic treatment is contraindicated, due in part to the elevated risk of HUS related to increased Shiga toxin (Stx) release associated with some antibiotics. Given the lack of effective strategies and the increasing number of STEC outbreaks, new treatment approaches are critically needed. In this study, we used an antimicrobial peptide wrwycr, previously shown to enhance STEC killing without increasing Stx production, in combination with antibiotic treatments. Checkerboard and time-kill assays were used to assess peptide wrwycr-antibiotic combinations for synergistic STEC killing. Cytotoxicity and real-time PCR were used to evaluate Stx production and *stx* expression, respectively, associated with these combinations. The synergistic combinations that showed rapid killing, no growth recovery and minimal Stx production were peptide wrwycr-kanamycin/gentamicin. Transmission electron microscopy revealed striking differences in bacterial cell morphology associated with various treatments. This study provides proof of principle for the design of an antibiotic-peptide wrwycr combination effective in killing STEC without enhancing release of Shiga toxins. It also offers a strategy for the repurposing of antibiotics for treatment of STEC infection.

## Introduction

Shiga toxin-producing *Escherichia coli* (STEC) is a major food- and water-borne pathogen that causes bloody diarrhea, hemorrhagic colitis and can lead to life-threatening sequelae including hemolytic uremic syndrome (HUS)^1–3,4^. This pathogen has been associated with numerous outbreaks and constitutes a serious global public health threat. Of the over 380 STEC serotypes, O157:H7 is the serotype most highly associated with outbreaks and severe disease sequelae in North America. Furthermore, the number of multi-state foodborne outbreaks linked to this serotype have been increasing, according to the Centers for Disease Control and Prevention (CDC) and the U.S. Food and Drug Administration (FDA). STEC infection typically begins with vomiting, watery diarrhea and abdominal cramping that progresses to bloody diarrhea. In the majority of people, the infection typically resolves within 7–10 days. However, in 5–7% of infected persons, the infection leads to a systemic, sometimes life-threatening complication known as HUS, a development which is mediated by the bacterial expression of Shiga toxins, Stx1 and Stx2, encoded by the *stx1* and *stx2* genes^5–8^. HUS is characterized by thrombocytopenia, hemolytic anemia and acute renal failure. Currently, treatment consists primarily of intravenous fluid volume expansion and supportive therapy^9–11^, while antimicrobials generally used to treat bacterial infections are often contraindicated. The reason for this counter-intuitive practice is that *in vitro* studies have shown that, at least for STEC O157, sublethal doses of different antibiotics and classes of antibiotics, including ampicillin, ciprofloxacin, ceftazidime, trimethoprim-sulfamethoxazole, furazolidone and the quinolones can promote the production and release of Shiga toxins that may enhance the risk of progression to HUS^5,12–15^. On the other hand, *in vitro* STEC treatment with macrolides, carbapenems, aminoglycosides, rifampin, rifaximin, and fosfomycin have been found to have either no effect on Stx production or to result in a decrease in Stx production^16,17^. Furthermore, a number of clinical studies have shown that patients on antibiotic therapy for STEC induced-hemorrhagic colitis have a higher risk of developing HUS^14^. However, the relevance of these findings is limited by small sample sizes in those studies and in some cases, by the advanced stage of illness of the patients. Other therapeutic treatments that are often used to treat gastrointestinal infections including antimotility agents, narcotics and non-steroidal anti-inflammatory medications are also not recommended for STEC infection. Given the increasing number of STEC outbreaks and HUS complications and the lack of available therapeutic strategies, there is a critical need for new approaches to the treatment of STEC infection. Recent studies looking at potential treatment strategies have identified forms of antibody therapy, plasmapheresis, zinc-based salts, small peptide molecules and probiotics^18–22^ but to date none have been adopted in clinical treatment of STEC infection.

We have previously reported that an antimicrobial peptide, wrwycr in its D isomer form, is able to significantly enhance acid-induced killing of all STEC seropathotypes associated with HUS and does so without increasing Stx release^23^. This peptide wrwycr has been shown to inhibit mechanisms of DNA damage repair and recombination that proceed through Holiday Junction (HJ) intermediates^24,25^. Further studies have expanded the list of potential intracellular bacterial targets of the peptide wrwycr, indicating that it causes cells to phenotypically starve for iron^26^. Consequently, wrwycr is a potent, broad-spectrum antimicrobial that is able to inhibit bacterial growth in a dose-dependent manner and as a D isomer should be resistant to intestinal proteases^23,27,28^.

We have also shown that this peptide wrwycr dramatically ameliorates *Citrobacter rodentium* infection in a mouse model of enterohemorrhagic *E. coli* (EHEC) infection, without disrupting the commensal flora profile^29^. Furthermore, repeated challenge does not increase resistance of the pathogen to the peptide wrwycr. Together, these studies suggest that the peptide wrwycr is a useful bacteriocidal agent that can potentiate gastric acid killing of the pathogen, making it a promising preventative agent.

However, we are also interested in pursuing whether the peptide wrwycr could serve as a treatment strategy for STEC O157:H7 infection. Our earlier work indicates that on its own, micromolar concentrations of the peptide wrwycr are not sufficient to eradicate all STEC O157:H7 and that minimum inhibitory concentrations (MIC) were relatively high. However, it is possible that if combined with a second antimicrobial/antibiotic, especially one that does not increase Stx, the peptide wrwycr may potentiate the activity of the second antimicrobial/antibiotic (or vice versa) and may do so at a lower combined dose of both peptide wrwycr and antibiotic. Simultaneously, the specificity of two different antimicrobial compounds coupled with a lower therapeutic dose may minimize the development of antimicrobial resistance arising from the use of either one. In this paper, we evaluate pairwise combinations of the peptide wrwycr with several antibiotics for synergistic activity determining the fractional inhibitory concentration index (FICI) using a standard checkerboard assay. The antibiotics selected include several that have been reported to have either no change or a decrease in Stx production allowing us to determine how the combination of each of these antibiotics with peptide wrwycr alters Stx production/release. We also evaluate two antibiotics that have been reported to increase Stx production/release to establish whether it is possible to find a combination of peptide wrwycr with these antibiotics that could produce a bactericidal effect without increasing Stx production. In this way, we may be able to explore the repurposing of antibiotics for treatment of STEC infection. Finally, we also examine the impact of peptide wrwycr on pathogen morphology and ultrastructure through transmission electron microscopy (TEM) of treated cells. The micrographs offer insights into the mechanisms of action of the peptide wrwycr and the impact of peptide wrwycr-antibiotic combinations on cellular integrity. This paper provides a proof of principle for the design of an antibiotic-peptide wrwycr combination that is effective in completely killing STEC without enhancing release of Shiga toxins.

## Results

### Antimicrobial activity of peptide wrwycr and selected antibiotics

We first determined the MIC values for the individual antimicrobials, including the peptide wrwycr and selected antibiotics (Table 1). The MIC value for the peptide wrwycr was 128 μg/mL against both STEC strains, 86–24 and EDL933. Antibiotics selected for this study include those reported to have either no change or a decrease in Stx production (gentamicin, kanamycin, meropenem) as well as two reported to cause an increase in Shiga toxin production (ciprofloxacin, chloramphenicol)^13,16,17^. MIC values determined for the selected antibiotics (Table 1) agree with previous reports for STEC O157:H7 strains^30^.

**Table 1:** MIC values for the peptide and selected antibiotics, as determined by a standard MIC assay. All assays were carried out in triplicate and on at least two different occasions.

Treatment	MIC ranges (μg/mL) for strain 86–24				MIC ranges (μg/mL) for strain EDL933			
	Min.	Max.	Median	Mode	Min.	Max.	Median	Mode
Peptide	128.00	256.00	128.00	128.00	64.00	256.00	128.00	128.00
Gentamicin	2.00	8.00	4.00	4.00	2.00	4.00	4.00	2–4
Kanamycin	4.00	16.00	8.00	8–16	4.00	16.00	8.00	8.00
Chloramphenicol	8.00	16.00	8.00	8.00	4.00	8.00	6.00	4–8
Ciprofloxacin	0.06	0.06	0.06	0.06	0.03	0.06	0.06	0.06
Meropenem	0.03	0.06	0.06	0.06	0.02	0.06	0.06	0.06

### Evaluating peptide wrwycr-antibiotic treatment combinations for synergistic effects

We next determined MIC values for various combinations of peptide with antibiotics and calculated FICI values to establish whether there was evidence of synergy in the combination treatment. We found synergistic FICIs for at least 4 of the 5 antibiotic-peptide wrwycr combinations for both strains STEC 86–24 and EDL933 (Table 2, Fig. S1 in the supplemental material). FICI values for both strains were remarkably similar. For STEC 86–24, combinations of peptide wrwycr and gentamicin produced FICI values of 0.19–0.50 (with ≤0.5 indicating synergy, as described in materials and methods) with combinations of peptide wrwycr between 8.00–32.0 μg/mL and gentamicin at 0.06–1.00 μg/mL (well below the gentamicin MIC of 4.00 μg/mL). In the case of kanamycin, FICI values ranging from 0.25–0.50 were achieved with peptide wrwycr ranging from 2.00–32.0 μg/mL and kanamycin from 0.13–4.00 μg/mL (well below the kanamycin MIC of 8.00–16.0 μg/mL). For chloramphenicol, FICI values of 0.28–0.50 were produced with combinations of peptide wrwycr (32.0 μg/mL) and chloramphenicol (0.25–2.00 μg/mL) (below the chloramphenicol MIC of 8.00 μg/Ml). Combinations of peptide wrwycr and ciprofloxacin produced FICI values of 0.31–0.50, where concentrations of peptide wrwycr were between 8.00–32.0 μg/mL (well below the peptide wrwycr MIC of 128 μg/mL) and ciprofloxacin concentrations were between 0.004–0.016 μg/mL (well below the ciprofloxacin MIC of 0.06 μg/mL). Finally, combinations of peptide wrwycr and meropenem produced an additive mean FICI of 0.51, with synergistic FICI values of 0.31–0.50 using a peptide wrwycr concentration of 32.0 μg/mL and 0.004–0.016 μg/mL meropenem which was well below the MIC value of 0.06 for meropenem alone. These results provide convincing evidence that several antibiotics potentiate the killing effect of the peptide wrwycr for both STEC strains and at substantially lower concentrations than the individual treatments.

**Table 2:** Fractional inhibitory concentration indices calculated for peptide antibiotic combinations that inhibited STEC strains 86–24 and EDL933. All assays were carried out in triplicate and on at least two different occasions.

Treatment	FICI Ranges		FICI Mean	Interaction
	Min.	Max.		
**86–24 strain**				
Gentamicin + Peptide	0.19	0.52	0.40	Synergistic
Kanamycin + Peptide	0.25	0.51	0.32	Synergistic
Chloramphenicol + Peptide	0.28	0.63	0.48	Synergistic
Ciprofloxacin + Peptide	0.31	0.53	0.46	Synergistic
Meropenem + Peptide	0.31	0.63	0.51	Additive
**EDL933 strain**				
Gentamicin + Peptide	0.16	0.50	0.30	Synergistic
Kanamycin + Peptide	0.16	0.51	0.32	Synergistic
Chloramphenicol + Peptide	0.28	0.75	0.49	Synergistic
Ciprofloxacin + Peptide	0.27	0.52	0.36	Synergistic
Meropenem + Peptide	0.31	0.63	0.50	Synergistic

To further investigate the impact of treatment combinations on STEC survival and the potential for recovery or development of resistance in STEC strain 86–24, we evaluated combinations of peptide wrwycr and antibiotic in time-kill assays over 24 hr (Table 3). The first section shows the time-kill results for MIC concentrations of the individual antimicrobials. The second section provides the time-kill results for subMIC concentrations of the individual antimicrobials. The next five sections show the time-kill results over 24 hr for synergistic combinations of the peptide wrwycr with each of the antibiotics at concentrations below their subMIC values. The log reduction in bacterial numbers is shown in the final right-hand column of Table 3. In all cases, with the exception of two combinations of chloramphenicol and peptide wrwycr, the synergistic combinations (with their corresponding FICI values reported in the second column) resulted in at least a 3-log reduction from the starting CFU/mL values, a decrease which is an accepted standard for bactericidal agents according to the CLSI guidelines, M26-A, vol. 19.

**Table 3:** Kinetics of STEC 86–24 killing using peptide, antibiotics and synergistic combinations. Data are expressed as means ± standard deviation. All assays were carried out in triplicate and on at least two different occasions.

Antimicrobial doses (µg/mL)^d^	FICI values	Viable cell count in specific time points over 24 h incubation in log_10_ CFU/mL ± SD^a^									TK^b^
		0 h	0.5 h	1 h	2 h	3 h	5 h	7 h	12 h	24 h	
**MIC Doses**											
PEP 128 (MIC)	---	6.0 ± 0.1	4.9 ± 0.1^**S**^	4.3 ± 0.1^**S**^	2.2 ± 0.2^**S**^	0.0 ± 0.0^**S**^	0.0 ± 0.0^**S**^	0.0 ± 0.0^**S**^	0.0 ± 0.0^**S**^	0.0 ± 0.0^**S**^	3.8|2h
GEN 4 (MIC)	---	6.0 ± 0.1	4.5 ± 0.4^**S**^	3.2 ± 0.1^**S**^	0.0 ± 0.0^**S**^	0.0 ± 0.0^**S**^	0.0 ± 0.0^**S**^	0.0 ± 0.0^**S**^	0.0 ± 0.0^**S**^	0.0 ± 0.0^**S**^	6.0|2h
KAN 16 (MIC)	---	6.0 ± 0.1	6.0 ± 0.0	5.2 ± 0.0^**S**^	3.8 ± 0.7^**S**^	0.7 ± 1.2^**S**^	0.0 ± 0.0^**S**^	0.0 ± 0.0^**S**^	0.0 ± 0.0^**S**^	0.0 ± 0.0^**S**^	5.3|3h
CHL 8 (MIC)	---	5.9 ± 0.1	6.0 ± 0.1	6.0 ± 0.0	5.9 ± 0.1^**S**^	5.8 ± 0.1^**S**^	5.7 ± 0.1^**S**^	5.6 ± 0.1^**S**^	5.6 ± 0.0^**S**^	5.4 ± 0.1^**S**^	No-TK
CIP 0.063 (MIC)	---	5.7 ± 0.1	6.0 ± 0.1	5.8 ± 0.1	3.1 ± 0.0^**S**^	1.3 ± 1.2^**S**^	0.7 ± 1.2^**S**^	0.0 ± 0.0^**S**^	0.0 ± 0.0^**S**^	0.0 ± 0.0^**S**^	4.4|3h
MER 0.063 (MIC)	---	5.8 ± 0.1	6.0 ± 0.1	5.1 ± 0.1^**S**^	2.7 ± 0.2^**S**^	0.7 ± 1.2^**S**^	0.7 ± 1.2^**S**^	0.7 ± 1.2^**S**^	0.0 ± 0.0^**S**^	0.0 ± 0.0^**S**^	3.1|2h
**SubMIC Doses**											
PEP 64 (subMIC)	---	6.0 ± 0.1	6.1 ± 0.0	6.1 ± 0.1	5.7 ± 0.0^**S**^	5.2 ± 0.1^**S**^	4.2 ± 0.1^**S**^	3.7 ± 0.2^**S**^	3.2 ± 0.1^**S**^	**4.9 ± 0.1**^**S**^	No-TK^c^
GEN 2 (subMIC)	---	6.0 ± 0.1	5.8 ± 0.1	4.8 ± 0.1^**S**^	0.7 ± 1.2^**S**^	0.7 ± 1.2^**S**^	**3.3 ± 0.1**^**S**^	**4.5 ± 0.1**^**S**^	**5.8 ± 0.1**^**S**^	**8.8 ± 0.2**^**S**^	5.3|2h^c^
KAN 8 (subMIC)	---	6.0 ± 0.1	6.1 ± 0.0	6.3 ± 0.1	5.4 ± 0.0^**S**^	2.4 ± 0.1^**S**^	2.4 ± 0.1^**S**^	1.4 ± 1.3^**S**^	0.7 ± 1.2^**S**^	**4.0 ± 0.1**^**S**^	3.6|3h^c^
CIP 0.031 (subMIC)	---	5.7 ± 0.1	6.2 ± 0.1	6.2 ± 0.1	5.1 ± 0.1^**S**^	4.2 ± 0.1^**S**^	3.7 ± 0.0^**S**^	3.2 ± 0.1^**S**^	**5.2 ± 0.1**^**S**^	**7.8 ± 0.1**^**S**^	No-TK^c^
MER 0.031 (subMIC)	---	5.8 ± 0.1	6.1 ± 0.1	5.9 ± 0.1	5.4 ± 0.0^**S**^	4.7 ± 0.1^**S**^	2.9 ± 0.1^**S**^	2.6 ± 0.2^**S**^	2.5 ± 0.1^**S**^	**3.9 ± 0.1**^**S**^	3.2|7h^c^
**Gentamycin + Peptide Combinations**											
GEN 0.50 + PEP 32	0.38	6.0 ± 0.1	3.3 ± 0.0^**S**^	0.0 ± 0.0^**S**^	0.0 ± 0.0^**S**^	0.0 ± 0.0^**S**^	0.0 ± 0.0^**S**^	0.0 ± 0.0^**S**^	0.0 ± 0.0^**S**^	0.0 ± 0.0^**S**^	6.0|1h
GEN 0.25 + PEP 32	0.31	6.0 ± 0.1	3.6 ± 0.1^**S**^	0.0 ± 0.0^**S**^	0.0 ± 0.0^**S**^	0.0 ± 0.0^**S**^	0.0 ± 0.0^**S**^	0.0 ± 0.0^**S**^	0.0 ± 0.0^**S**^	0.0 ± 0.0^**S**^	6.0|1h
GEN 0.13 + PEP 32	0.28	6.0 ± 0.1	5.0 ± 0.1^**S**^	2.8 ± 0.8^**S**^	0.0 ± 0.0^**S**^	0.0 ± 0.0^**S**^	0.0 ± 0.0^**S**^	0.0 ± 0.0^**S**^	0.0 ± 0.0^**S**^	**3.4 ± 0.1**^**S**^	3.2|1h^c^
GEN 0.50 + PEP 16	0.25	6.0 ± 0.1	4.6 ± 0.3^**S**^	2.7 ± 0.6^**S**^	0.7 ± 1.2^**S**^	0.7 ± 1.2^**S**^	0.0 ± 0.0^**S**^	0.0 ± 0.0^**S**^	0.0 ± 0.0^**S**^	0.0 ± 0.0^**S**^	3.3|1h
GEN 0.25 + PEP 16	0.19	6.0 ± 0.1	5.7 ± 0.1	4.7 ± 0.1^**S**^	3.0 ± 0.0^**S**^	2.4 ± 0.1^**S**^	1.6 ± 1.4^**S**^	2.3 ± 0.2^**S**^	**3.0 ± 0.1**^**S**^	**4.2 ± 0.1**^**S**^	3.0|2h^c^
**Kanamycin + Peptide Combinations**											
KAN 2 + PEP 32	0.38	6.0 ± 0.1	5.0 ± 0.1^**S**^	2.7 ± 0.1^**S**^	0.7 ± 1.2^**S**^	0.0 ± 0.0^**S**^	0.0 ± 0.0^**S**^	0.0 ± 0.0^**S**^	0.0 ± 0.0^**S**^	0.0 ± 0.0^**S**^	3.3|1h
KAN 4 + PEP 16	0.38	6.0 ± 0.1	6.0 ± 0.0	3.9 ± 0.0^**S**^	2.5 ± 0.1^**S**^	0.0 ± 0.0^**S**^	0.0 ± 0.0^**S**^	0.0 ± 0.0^**S**^	0.0 ± 0.0^**S**^	0.0 ± 0.0^**S**^	3.5|2h
KAN 4 + PEP 8	0.31	6.0 ± 0.1	6.1 ± 0.0	5.0 ± 0.1^**S**^	3.5 ± 0.0^**S**^	2.8 ± 0.1^**S**^	0.0 ± 0.0^**S**^	0.0 ± 0.0^**S**^	0.0 ± 0.0^**S**^	0.0 ± 0.0^**S**^	3.2|3h
KAN 1 + PEP 32	0.31	6.0 ± 0.1	5.9 ± 0.1	4.5 ± 0.0^**S**^	2.7 ± 0.2^**S**^	0.7 ± 1.2^**S**^	0.0 ± 0.0^**S**^	0.0 ± 0.0^**S**^	0.0 ± 0.0^**S**^	**3.9 ± 0.1**^**S**^	3.3|2h^c^
KAN 4 + PEP 4	0.28	6.0 ± 0.1	6.1 ± 0.0	4.5 ± 0.0^**S**^	2.7 ± 0.2^**S**^	0.0 ± 0.0^**S**^	0.0 ± 0.0^**S**^	0.0 ± 0.0^**S**^	0.0 ± 0.0^**S**^	**3.8 ± 0.0**^**S**^	3.3|2h^c^
KAN 2 + PEP 16	0.25	6.0 ± 0.1	5.7 ± 0.2	5.0 ± 0.1^**S**^	3.0 ± 0.0^**S**^	0.0 ± 0.0^**S**^	0.0 ± 0.0^**S**^	0.0 ± 0.0^**S**^	0.0 ± 0.0^**S**^	**5.0 ± 0.1**^**S**^	3.0|2h^c^
**Chloramphenicol + Peptide Combinations**											
CHL 2 + PEP 32	0.50	5.9 ± 0.1	5.7 ± 0.1	5.4 ± 0.0	4.8 ± 0.0^**S**^	4.4 ± 0.1^**S**^	4.2 ± 0.0^**S**^	3.9 ± 0.1^**S**^	3.1 ± 0.1^**S**^	2.5 ± 0.2^**S**^	3.4|24h
CHL 1 + PEP 32	0.38	5.9 ± 0.1	5.9 ± 0.0	5.8 ± 0.1	5.5 ± 0.0^**S**^	5.3 ± 0.1^**S**^	4.8 ± 0.1^**S**^	4.1 ± 0.1^**S**^	**4.3 ± 0.1**^**S**^	**4.7 ± 0.0**^**S**^	No-TK^c^
CHL 0.5 + PEP 32	0.31	5.9 ± 0.1	6.0 ± 0.0	5.9 ± 0.0	5.3 ± 0.1^**S**^	4.4 ± 0.0^**S**^	4.3 ± 0.0^**S**^	4.3 ± 0.1^**S**^	**4.4 ± 0.1**^**S**^	**4.7 ± 0.0**^**S**^	No-TK^c^
**Ciprofloxacin + Peptide Combinations**											
CIP 0.016 + PEP 32	0.50	5.7 ± 0.1	6.0 ± 0.1	5.9 ± 0.1	5.2 ± 0.1^**S**^	4.2 ± 0.0^**S**^	1.3 ± 1.2^**S**^	1.3 ± 1.2^**S**^	0.0 ± 0.0^**S**^	**1.3 ± 1.2**^**S**^	4.4|5h^c^
CIP 0.008 + PEP 32	0.38	5.7 ± 0.1	5.9 ± 0.1	6.0 ± 0.1	5.3 ± 0.1^**S**^	4.5 ± 0.1^**S**^	2.6 ± 0.1^**S**^	1.5 ± 1.3^**S**^	0.0 ± 0.0^**S**^	**3.2 ± 0.1**^**S**^	3.1|5h^c^
CIP 0.004 + PEP 32	0.31	5.7 ± 0.1	6.0 ± 0.2	5.8 ± 0.1	5.7 ± 0.1^**S**^	5.3 ± 0.1^**S**^	3.3 ± 0.0^**S**^	3.1 ± 0.1^**S**^	2.5 ± 0.1^**S**^	**6.7 ± 0.0**^**S**^	3.2|12h^c^
CIP 0.016 + PEP 16	0.38	5.7 ± 0.1	6.0 ± 0.1	6.0 ± 0.2	5.1 ± 0.0^**S**^	4.1 ± 0.0^**S**^	3.1 ± 0.0^**S**^	2.7 ± 0.1^**S**^	0.0 ± 0.0^**S**^	**2.5 ± 0.2**^**S**^	3.0|7h^c^
**Meropenem + Peptide Combinations**											
MER 0.016 + PEP 32	0.50	5.8 ± 0.1	6.1 ± 0.1	6.1 ± 0.1	5.7 ± 0.1^**S**^	5.2 ± 0.0^**S**^	4.7 ± 0.0^**S**^	4.7 ± 0.1^**S**^	0.0 ± 0.0^**S**^	0.0 ± 0.0^**S**^	5.8|12h
MER 0.008 + PEP 32	0.38	5.8 ± 0.1	6.0 ± 0.1	6.1 ± 0.1	5.7 ± 0.0^**S**^	5.3 ± 0.1^**S**^	4.7 ± 0.0^**S**^	4.8 ± 0.0^**S**^	1.5 ± 1.3^**S**^	**3.3 ± 0.1**^**S**^	4.3|12h^c^

^a^Evaluation of time-kill assay in bacterial cultures treated with peptide, antibiotic and synergistic combinations. Data are expressed in log_10_ CFU/mL as means ± standard deviations of two independent experiments in triplicate. ^**S**^One-way ANOVA and Tukey’s test were used to determine means statistically significant compared to the no-antimicrobial control (p < 0.05).

^b^TK (Time-kill) = log decrease|bactericidal effect hr (at least 3-log_10_ reduction in the original inoculum according CLSI guidelines, M26-A, vol. 19).

^c^Signs of bacterial recovery from subMIC treatments and the lowest FICI values (data are shown in bold).

^d^PEP, peptide; GEN, gentamicin; KAN, kanamycin; CHL, chloramphenicol; CIP, ciprofloxacin; MER, meropenem.

For synergistic gentamicin-peptide wrwycr treatment combinations, killing was more rapid than any of the individual treatments, provided that FICI values were 0.31–0.38 (*i.e.* when peptide wrwycr concentrations were 32.0 μg/mL). When FICI values were lower than 0.28 (*i.e.* when peptide wrwycr concentrations were lower than 32.0 μg/mL), the rate of killing was reduced at the lowest combined concentration and there was some evidence of growth recovery after 7–12 hours with one exception (combination P:16 + G:1 μg/mL; FICI 0.25).

For kanamycin-peptide wrwycr treatments, killing was more rapid and effective than that for the individual MIC or subMIC treatments, when FICI values were 0.31–0.38 when peptide wrwycr concentrations were 16.0–32.0 μg/mL and the kanamycin concentrations were 2.00–4.00 μg/mL. For combinations that generated lower FICI values ranging from 0.31–0.25 and when the peptide wrwycr and kanamycin concentrations were below 32.0 μg/mL and 4.00 μg/mL respectively, there was some evidence of growth recovery after 12 hr.

For chloramphenicol, all combinations identified by checkerboard as synergistic showed killing rates similar to the same concentration of peptide wrwycr and there was evidence of some recovery after 7–12 hr treatment. These data indicate the bacteriostatic rather than bactericidal activity of chloramphenicol on its own. Interestingly, for the subMIC combination of chloramphenicol with peptide wrwycr of P:32 + C:2 μg/mL (FICI 0.50), STEC killing was enhanced 3.4 log fold, achieving a bactericidal effect in marked contrast to the MIC chloramphenicol treatment alone.

For ciprofloxacin, in the case of all four combinations identified as synergistic, the rate of killing was relatively slow but still more effective than corresponding subMIC concentrations of the individual antimicrobials. However, there was evidence of growth recovery after 12 hr treatment.

Similarly, for meropenem, the combinations identified as synergistic showed a similar rate of killing relative to individual subMIC treatments. When the FICI value was lower than 0.50 (i.e. when the meropenem concentration within the combination was 0.008 μg/mL), there was evidence of some recovery of growth after 12hr treatment.

**Figure 1:**
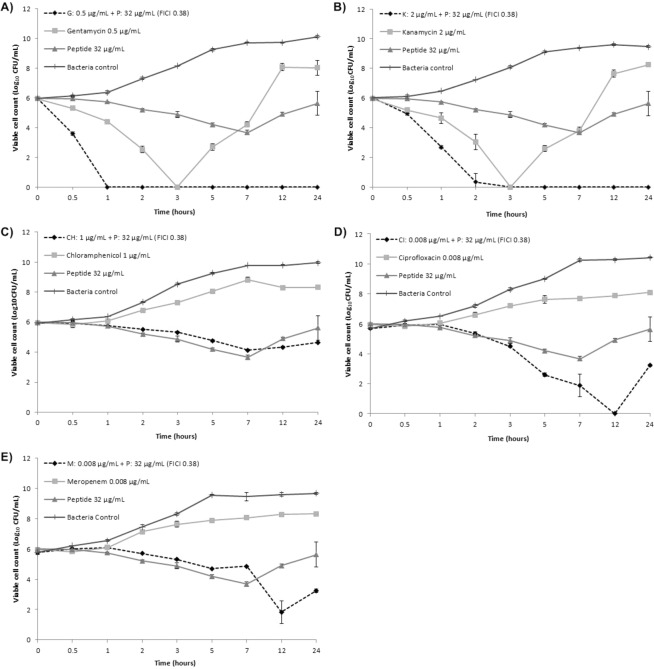
STEC survival over the course of a 24 hour killing assay as determined by serial plate count assay and expressed as viable counts per mL of culture. Each graph provides survival curves for STEC treatment with the subMIC concentrations of each of antibiotic or peptide or antibiotic-peptide combination relative to growth of untreated STEC (bacterial control). In all cases, the peptide dose was 32 µg/mL and the FICI value for peptide-antibiotic combinations was 0.38. Time kill curves for peptide alone or in combination with (**A**) Gentamicin (0.5 µg/mL) (**B**) Kanamycin (2.0 µg/mL) (**C**) Chloramphenicol (1.0 µg/mL) (**D**) Ciprofloxacin (0.008 µg/mL) (**E**) Meropenem (0.008 µg/mL). All assays were carried out in triplicate and on at least two different occasions.

In Fig. 1, we have highlighted specific time-kill curves showing combinations of peptide wrwycr with each of the antimicrobials relative to the time-kill curves for the individual antimicrobials at the same subMIC concentrations. In each case, we used a consistent subMIC peptide wrwycr concentration of 32.0 μg/mL and a FICI value of 0.38 so as to provide relevant comparisons. Figure 1 clearly shows that the synergistic combinations of peptide wrwycr with gentamicin (Fig. 1A) or peptide wrwycr with kanamycin (Fig. 1B) are significantly more effective at bacterial killing than the individual antimicrobials at the same subMIC concentrations as in the synergistic combinations. For chloramphenicol, the killing curve clearly confirms the bacteriostatic rather than bacteriocidal activity of chloramphenicol on its own and reveals that the combination was no more effective than the peptide wrwycr on its own (Fig. 1C). For ciprofloxacin and meropenem, the combinations with peptide wrwycr showed similar or slightly improved rates of killing relative to the individual antimicrobials (Fig. 1D,E).

Taken together, the results of the killing assays provide valuable insight into the subMIC peptide wrwycr-antibiotic combination treatments and indicate that the most effective synergistic combinations,* i.e.* those that show rapid killing and no growth recovery, are combinations of peptide wrwycr with either kanamycin or gentamicin. These results also demonstrate the critical importance of complementing standard checkerboard assays with killing assays over a prolonged time course to permit the detection of growth recovery. This is especially important for combinations that have been identified as synergistic by checkerboard assay.

Since the peptide-gentamicin combination (P:16 + G:1 µg/mL) produced a synergistic response for both strains STEC 86–24 and EDL933, we extended this evaluation to a panel of strains, including K12 (control) and 14 additional clinical STEC isolates including both O157 and nonO157 strains and representing all 5 seropathotypes previously characterized by Karmali *et al*.^31^. Of the total of 15 strains tested, the majority were susceptible to all three treatments (peptide, gentamicin, or peptide-gentamicin), indicating low/no resistance of clinical isolates to peptide (alone or in combination) (Table S1 in the supplemental section). Results also revealed that while there was variation in susceptibility to individual and combination treatment across the strains tested, it was clear that all strains were susceptible to the P:16 + G:1 combination that was shown to be synergistic in 86–24.

### Effect of peptide wrwycr-antibiotic combinations on Stx release, production and expression

We then sought to determine whether the peptide wrwycr-antibiotic combination treatments altered the production of active Stx. To do so, we used the well-established Vero cell cytotoxicity assay to determine the extent of Stx-mediated cytotoxicity resulting from various synergistic combination treatments relative to the individual antimicrobial treatments. In each case, we assessed the level of Vero cell cytotoxicity resulting from each of the secreted Stx (released) and periplasmic Stx (produced but not released) extracts produced by STEC 86–24. Based on the time-kill assay results, we chose to evaluate cytotoxicity levels at 6 hr of treatment, the time typically associated with maximum pathogen killing and 15 hr of treatment, the time where STEC growth recovery was occasionally noted during the time kill assays. It should be noted that STEC 86–24 only produces Stx2. The standard curve showing levels of Vero cell cytotoxicity induced by various concentrations of purified Stx2 is provided in Fig. S2 in the supplemental material.

**Figure 2:**
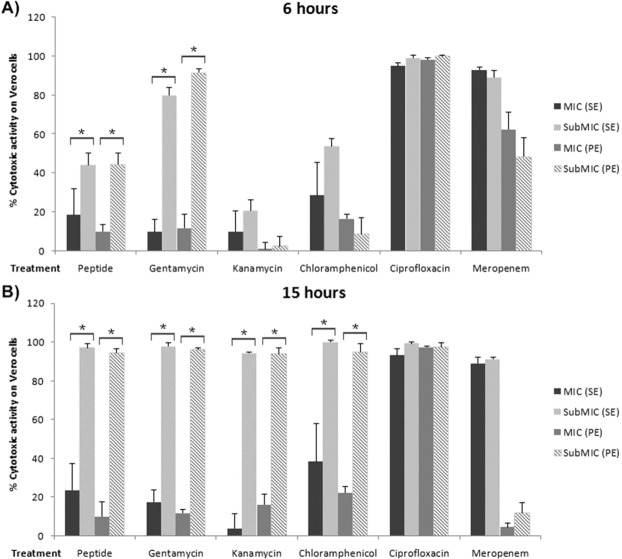
Vero cell cytotoxicity levels with secreted (SE) and seriplasmic (PE) lysates from STEC 86–24 following treatment with peptide or antibiotic after (**A**) 6 hr and (**B**) 15 hr. Antimicrobial doses included both MIC and subMIC doses: peptide (128 µg/mL and 64 µg/mL), gentamicin (4 µg/mL and 2 µg/mL), kanamycin (16 µg/mL and 8 µg/mL), chloramphenicol (8 µg/mL and 4 µg/mL), ciprofloxacin (0.063 µg/mL and 0.031 µg/mL) and meropenem (0.063 µg/mL and 0.031 µg/mL). All assays were carried out in triplicate and on at least two different occasions. Data bars represent means ± standard deviation. *Significantly different from subMIC treatment; *P* < 0.05; Unpaired student’s test with Welch’s correction.

In the first set of experiments, we compared levels of cytotoxicity resulting from STEC treatment with MIC versus subMIC concentrations of each of the antimicrobial (Fig. 2). Since the synergistic combination treatments all consisted of subMIC concentrations of both peptide wrwycr and antibiotic, it was important to evaluate and compare the levels of cytotoxicity associated with MIC versus subMIC levels for individual antimicrobials and then to compare cytotoxicity levels for the subMIC combination treatments. Figure 2 shows profound differences in cytotoxicity levels at both time points for MIC versus subMIC (Fig. 2A,B) treatments with each of the antimicrobials for both secreted (SE) and periplasmic (PE) extracts. In most cases, significantly higher levels of cytotoxicity were associated with individual subMIC antimicrobial treatments. Treatment of STEC with MIC concentrations of peptide wrwycr resulted in 10–24% Vero cell death for both 6 and 15 hr treatments, which is consistent with relatively low levels of Stx release for both secreted and periplasmic extracts, as previously reported (12). However, at subMIC concentrations of peptide wrwycr alone, cytotoxicity levels were dramatically increased, ranging from 42–98% Vero cell death, depending on the time point selected. Similarly, STEC treatment with MIC concentrations of gentamicin or kanamycin resulted in low levels of Vero cell death, both from secreted or periplasmic Stx (gentamicin: 10–17%; kanamycin: 1–17%), but showed significant increases in cytotoxicity for subMIC treatments, in some cases reaching almost 100%. Treatment with MIC concentrations of chloramphenicol, which has been reported to trigger increased Stx production/release, was found to generate 28–38% cell death via secreted Stx and 16–22% cell death through periplasmic Stx extracts. By contrast, treatment with subMIC chloramphenicol showed significant increases in cytotoxicity for the 6 hr secreted extract and both 15 hr extracts. STEC treatment with either MIC or subMIC concentrations of ciprofloxacin, known to trigger increased Stx release, resulted in very high levels of Vero cell death (93–98%) from each of secreted or periplasmic Stx at both time points. Finally, meropenem MIC treatment of STEC resulted in variable levels of cell death with higher levels of cell death produced by secreted Stx (89–94%) relative to periplasmic Stx extracts (4–62%). Similar levels of cytotoxicity were generated by treatment with subMIC levels of meropenem.

**Figure 3:**
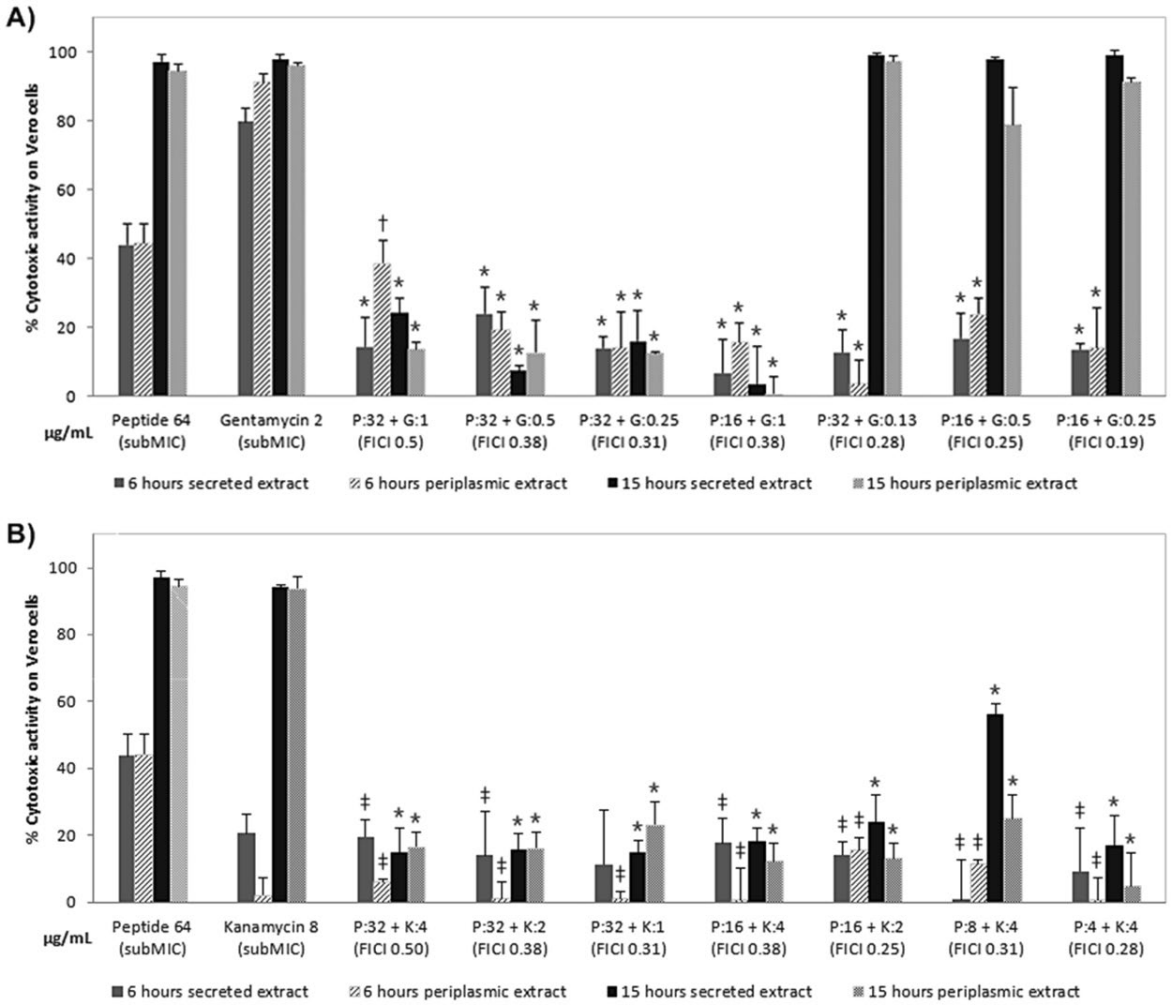
Vero cell cytotoxicity levels with secreted and periplasmic lysates from STEC 86–24 following treatment with peptide or antibiotic or peptide-antibiotic combinations after 6 hr and 15 hr. (**A**) with subMIC peptide-gentamicin combinations with specified FICI values (**B**) with subMIC peptide-kanamycin combinations with specified FICI values. All assays were carried out in triplicate and on at least two occasions. Data bars represent means ± standard deviation. *Significantly different from both individual treatments (peptide or antibiotic); ^†^Significant different from antibiotic treatment. ^‡^Significantly different from peptide treatment; *P *< 0.05; One-way ANOVA with post hoc Tukey comparisons.

Next, the cytotoxicity levels mediated by subMIC peptide wrwycr-antibiotic combinations treatments were tested (Figs. 3 and S3 in the supplemental material), and in some cases yielded strikingly different data than that for the individual treatments (Fig. S3A in the supplemental material).

For gentamicin, treatment with subMIC antibiotic-peptide wrwycr synergistic combinations with FICI values between 0.31–0.50 showed significantly lower Stx-mediated cytotoxicity with either secreted or periplasmic Stx extracts for both time points relative to individual subMIC antimicrobial treatments (Fig. 3A)^13^. However, for combination treatments with FICI values of 0.19–0.28 where we noted some bacterial recovery after 7–12 hr of treatment in the time-kill assay, there was an increase in cytotoxicity associated with both secreted and periplasmic Stx at the 15 hr treatment time. These data suggest that gentamicin-peptide wrwycr synergistic combinations with FICI values between 0.31–0.50 provide optimal pathogen killing while minimizing production and release of Stx.

Similarly, for kanamycin, there was a significant decrease relative to the individual subMIC treatments in the levels of cytotoxicity associated with either secreted or periplasmic extracts at both times points across all synergistic antimicrobial combinations with one exception (Fig. 3B). The combination treatment with a FICI value of 0.31 generated increased Stx production at the 15 hr time point. These results suggest that kanamycin-peptide wrwycr combinations, with the one exception noted, are effective against pathogen killing while minimizing Stx-mediated cytotoxicity.

For chloramphenicol, combinations with FICI values of 0.38–0.50 produced less cytotoxicity for both secreted and periplasmic extracts at both time points relative to the subMIC individual treatments (Fig. S3A in the supplemental material). However, combinations with lower FICI values (0.31) resulted in more Stx production/release (Fig. S3A in the supplemental material) than the MIC chloramphenicol treatment on its own (Fig. 2A,B). These data are also consistent with evidence of bacterial recovery that occurred after 7–12 hours of treatment at this lower FICI subMIC combination (Table 3).

For ciprofloxacin, all synergistic combinations generated high levels of cytotoxicity (79–100% cytotoxicity) across both extracts and both time points (Fig. S3B in the supplemental material). This was consistent with the levels of cytotoxicity seen for the subMIC ciprofloxacin treatment alone and demonstrated that at least in the case of this antibiotic, subMIC combinations with peptide wrwycr were not able to generate a more favourable outcome.

For meropenem, the results showed considerable variability. At a synergistic combination with a FICI value of 0.50, Stx-mediated cytotoxicity was similar to that for subMIC treatment of meropenem alone (Fig. S3C in the supplemental material). However, at a FICI value lower that 0.50, there was an increase in cytotoxicity across time points and extracts, but particularly so for the 15-hr periplasmic extract which may be associated with the bacterial recovery seen after 7–12 hr of this combination treatment. Interestingly, there was also an increase in Stx production in the additive combination with a FICI value of 0.75, particularly in the periplasmic extract. These results suggest that synergistic or additive combinations of meropenem and peptide wrwycr, while effective in pathogen killing, do not minimize Stx production and release.

Finally, we assessed changes in expression of the *stx2* gene in STEC 86–24 by real-time PCR for all treatment combinations (Fig. S4 in the supplemental material). The real-time results are consistent with the cytotoxicity results in Figs. 2 and 3.

These data provide evidence that subMIC combinations of peptide wrwycr-gentamicin and peptide wrwycr-kanamycin result in significantly lower levels of cytotoxicity than the individual treatments, suggesting that a lower therapeutic dose can enhance STEC killing without increasing Stx production/release. Secondly, these data provide valuable new insights into Stx-mediated cytotoxicity by tracking each of secreted (released) and periplasmic (produced not released) Stx extracts at two distinct time points. Evaluating both secreted and periplasmic Stx-mediated cytotoxicity provides a more detailed picture of potential levels of cytotoxicity and more relevant data than transcriptional results alone. Furthermore, multiple time points improves on previous studies that provide single measures of Stx-mediated cytotoxicity, with some studies focusing on the 4–6 hr treatment time frame and others looking only at later time points.

Since the peptide wrwycr-gentamicin combination treatment (P:16 + G:1 µg/mL) produced a synergistic response for STEC 86–24 with minimal associated cytotoxicity, we evaluated cytotoxicity levels for this same combination treatment for EDL933 relative to no treatment or treatment with peptide or gentamicin alone. While we saw higher levels of cytotoxicity for secreted extracts from EDL933, we found that the combination treatment resulted in less cytotoxicity in response relative to no treatment or treatment with either peptide or gentamicin (Fig. S5 in the supplemental data). We also evaluated the cytotoxicity levels of secreted extracts from 16 STEC strains treated with the peptide wrwycr-gentamicin (P:16 + G:1 µg/mL) combination. These strains included O157 and nonO157 strains and represented all 5 seropathotypes^31^ as well as K12 (the no toxin control). Results revealed variation in the levels of cytotoxicity across the strains (Fig. S6 in the supplemental section). This is likely related to high levels of variability in strain-to-strain cytotoxic potential, but suggests that the concentrations of combination treatments may require strain-specific titration to maximize effectiveness and minimize associated levels of cytotoxicity.

### Impact of peptide wrwycr and antibiotic treatment on bacterial cell ultrastructure

**Figure 4:**
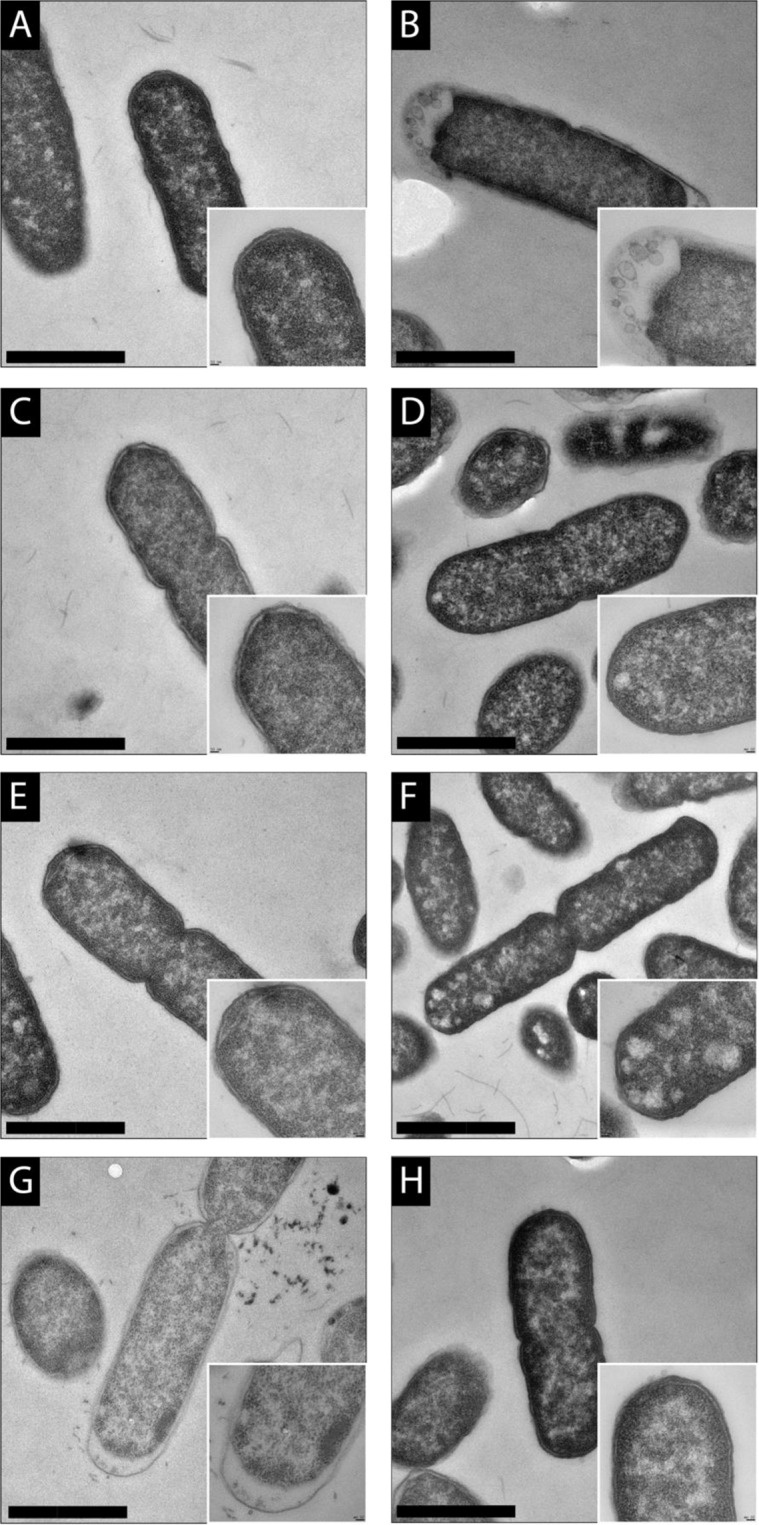
TEM micrographs of resin-embedded thin sections of STEC 86–24 following treatments with (**A**) no treatment (control) (**B**) peptide (128 μg/mL) (**C**) kanamycin (16 μg/mL) (**D**) gentamicin (4 μg/mL) (**E**) kanamycin (4 μg/mL) and peptide (8 μg/mL) (F) kanamycin (4 μg/mL) and peptide (4 μg/mL) (**G**) gentamicin (0.25 μg/mL) and peptide (32 μg/mL) (**H**) gentamicin (1 μg/mL) and peptide (16 μg/mL). Scale = 1 μm. These images are representative of images collected on two different occasions.

TEM was used to visualize physical changes that occurred at the cellular level when STEC 86–24 was treated with each of peptide wrwycr, kanamycin or gentamicin alone or in peptide wrwycr-antibiotic combinations. These treatments were selected for TEM evaluation because they generated effective synergistic combinations with minimal levels of Stx-mediated cytotoxicity. Figure 4 provides representative micrographs of resin-embedded thin sections that show the morphology and ultrastructure of the cells and reveals striking differences in bacterial cell morphology associated with various antimicrobial treatments. Relative to the no treatment control (Fig. 4A), treatment with the MIC concentration of peptide wrwycr (128 μg/mL) revealed an enlarged periplasmic space at the cell pole, enclosing a number of membrane-bound structures (Fig. 4B). Treatment with the MIC peptide wrwycr dose appears to primarily affect the cell poles and potential division sites. This could suggest that the peptide wrwycr works on curved portions of the cell with the outer membrane being compromised. The circular bodies are likely portions of the inner membrane and are similar to that previously observed with treatment with polymyxin B-nonapeptide.

Treatment with kanamycin (16.0 μg/mL) produced very few and minor disruptions at the poles with no obvious accumulation of membrane structures or expanded periplasm (Fig. 4C). Treatment with gentamicin (4.00 μg/mL; Fig. 4D) also produced little notable ultrastructural changes from untreated cells. Kanamycin (4.00 μg/mL) in combination with peptide wrwycr (8.00 ug/mL; FICI 0.31) resulted in some expansion of the periplasm and an accumulation of membrane structures at the cell poles (Fig. 4E). These changes were again less severe than seen with the peptide wrwycr alone at 128 μg/mL (Fig. 4B). Kanamycin (4.00 μg/mL) with a lower concentration of peptide wrwycr (4.00 μg/mL) resulted in less severe morphological changes, but typically had an accumulation of electron-light regions near the poles (Fig. 4F). Treatment with a low dose of gentamicin (0.25 μg/mL) combined with a high concentration of peptide wrwycr (32.0 μg/mL) produced similar structures seen with the peptide wrwycr alone, with an enlarged, elaborate periplasm at the poles and numerous membrane structures noted (Fig. 4G). Gentamicin concentrations (1.00 μg/mL) with a lower concentration of peptide wrwycr (16.0 μg/mL) produced only minor perturbations at the cell poles (Fig. 4H).

## Discussion

Recently, there has been significant interest in the possibility of repurposing antibiotics through the development of antibiotic combinations that generate synergistic effects at lower combined doses of the individual antibiotics. The potential benefits of this approach are enhanced antimicrobial activity and a decreased risk of antimicrobial resistance. Here, we provide a proof of principle evaluation of the effectiveness of treatments of the STEC O157:H7 strain 86–24 using different combinations of antibiotics with the novel antimicrobial peptide wrwycr. We found synergistic effects for 4 of the 5 antibiotic-peptide wrwycr combinations, where the dose required to achieve the synergistic effect was well below the individual MIC values. These results, which were the same for both O157:H7 strains 86–24 and EDL933, provide convincing evidence that the combinations potentiate the activity of the individual antimicrobials and at substantially lower doses than the individual treatments.

We evaluated the impact of treatment combinations on STEC 86–24 survival and the potential for recovery or development of resistance to individual and combined antimicrobial treatments. The gentamicin-peptide wrwycr and kanamycin-peptide wrwycr combinations yielded effective synergistic combinations with rapid killing and, depending on the combinations chosen, no evidence of recovery. It is interesting to note that the two most effective synergistic peptide wrwycr-antibiotic combinations for STEC 86–24 involve antibiotics that inhibit protein synthesis. This may be significant in terms of potentiation of peptide wrwycr mechanism of action. The peptide wrwycr-kanamycin and peptide wrwycr-gentamicin combinations provide several benefits over treatment with any of these agents on their own. Treatment with either kanamycin or gentamicin at MIC doses are associated with increased toxicity which would be minimized at the low dose required for the synergistic combination with peptide wrwycr. Furthermore, there are numerous reports of STEC resistance to both kanamycin and gentamicin^32–34^ that could be circumvented by using a peptide wrwycr-antibiotic combination treatment.

For the other antibiotics tested, it was possible to achieve combinations that would be defined as synergistic by checkerboard assay. However, when we examined these combinations using time-kill assays, where we were able to track growth recovery for STEC 86–24 over a longer period of time, we found that these “synergistic” combinations show evidence of growth recovery, challenging the definition of synergy based on checkerboard assays alone. These results demonstrate the critical importance of complementing checkerboard assays with killing assays over a prolonged time course. Taken together, the results of the killing assays provide valuable insight into the subMIC peptide wrwycr-antibiotic combination treatments and indicate that the most effective synergistic combinations, those that show rapid killing and no growth recovery, are combinations of peptide wrwycr with either kanamycin or gentamicin.

Our results also indicated that while the gentamicin:peptide combination was synergistically effective for the two STEC O157:H7 strains 86–24 and EDL933, there was considerable variability in the response of other STEC strains to this combination treatment. However, it was clear that all STEC strains tested were susceptible to combination treatment, and in many cases more susceptible than to individual treatments alone. The variability of these results suggest that strain-specific titration would be needed to fully optimize the combination dose for different STEC strains.

The level of cytotoxicity and Stx production associated with antibiotic treatment is an important consideration for the treatment of STEC infection. Our results show significant differences in cytotoxicity levels of STEC 86–24 for MIC versus subMIC treatments with each of the antimicrobials, with higher levels of cytotoxicity associated with subMIC individual antimicrobial treatments relative to the peptide wrwycr-antibiotic combination treatments in almost all cases. These findings are consistent with reports that Stx release and production is enhanced in subMIC antibiotic treatments where the SOS response is triggered^35–37^. The major concern with antibiotic treatment of STEC infection is the associated increased risk of developing HUS, which has been linked to subMIC (rather than MIC) concentrations of several antibiotics. Consequently, it was important to evaluate combinations of peptide wrwycr-antibiotics where the individual doses are low, but the combinations are synergistic and effective at killing STEC. In the case of the peptide wrwycr-antibiotic combinations, the synergistic gentamicin-peptide wrwycr combinations that showed optimal, rapid STEC 86–24 killing (those with FICI values between 0.31–0.38) also minimized levels of cytotoxicity associated with both secreted and periplasmic Stx at all time points tested. The results indicate that the combination treatment for STEC 86–24 (as well as for EDL933) was both more effective at killing and generated less Stx production and release than treatments of peptide wrwycr or gentamicin alone. Similar results were achieved with synergistic kanamycin-peptide wrwycr combinations where significant decreases in cytotoxicity levels were noted for both secreted and periplasmic extracts from STEC 86–24 at both 6 hr and 15 hr time points with one exception. The combination treatment with a FICI value of 0.31 generated increased Stx production at the 15 hr time point. This provides a cautionary note for the consideration of combinations with lower FICI values that exhibit some growth recovery as a treatment option for STEC infection. However, administration of treatment multiple times per day to reduce pathogen recovery could resolve this challenge. For chloramphenicol, the combinations with FICI values of 0.50–0.38 produced less cytotoxicity for both secreted and periplasmic extracts at both time points relative to the subMIC individual treatments but the chloramphenicol-peptide wrwycr combination with FICI value of 0.31 resulted in more Stx production/release than the MIC chloramphenicol treatment on its own. Previous *in vitro* studies found that chloramphenicol did not influence Stx transcription^17,38^. Our results show a lower cytotoxicity (<40%) for both secreted and periplasmic extracts of chloramphenicol-treated STEC 86–24. More importantly, we also note the bactericidal effect of the chloramphenicol-peptide wrwycr combination (FICI 0.50) which shows an optimal killing (a reduction of 3.4 log fold (>99.9%) of the original inoculum) has no evidence of bacterial recovery. This may provide a useful strategy for antimicrobial treatment for STEC infection. For ciprofloxacin, all synergistic combinations generated high levels of cytotoxicity for both extracts at both time points. Finally, the results suggest that synergistic or additive combinations of meropenem with peptide wrwycr, while effective in pathogen killing, do not minimize Stx production and release. When we evaluated the impact of the synergistic gentamicin-peptide wrwycr combination on cytotoxicity levels of a panel of STEC strains, it was apparent that there was considerable variability across the strains, emphasizing the importance of tailoring the combination treatment for different strains and specifically evaluating these treatments relative to no treatment or to individual treatments.

Our data also provide valuable new insights into Stx cytotoxicity associated with antimicrobial treatments through tracking both the secreted (released) and periplasmic (produced not released) Stx extracts at two distinct time points. Evaluating both secreted and periplasmic Stx-mediated cytotoxicity provides a clearer picture of potential levels of cytotoxicity expected and also more relevant data than transcriptional results. Evaluation of STEC ultrastructure by TEM provided valuable insights into the effects of each antimicrobial treatment compared with antibiotics used in combination with peptide wrwycr. The images suggest that the synergistic effect between peptide wrwycr and either kanamycin or gentamicin is likely associated with enhanced disruption of the cell membrane, which could potentially increase access of the antibiotic to the inside the bacterial cell, thereby rendering the lower antibiotic concentrations more effective. Interestingly, the synergistic combinations of either kanamycin plus peptide wrwycr or gentamicin plus peptide wrwycr appeared to produce more modest changes including some periplasmic swelling and membrane bound bodies but certainly much less than that noted with the peptide wrwycr alone.

In conclusion, this paper provides an important evaluation of different antimicrobial treatments on *in vitro* STEC survival and associated Stx cytotoxicity. It also provides evidence of highly effective synergistic combinations of peptide wrwycr with each of kanamycin or gentamicin that show rapid killing with no growth recovery and minimal Stx-associated cytotoxicity. Finally, this study provides proof of principle for the design of an antibiotic-peptide wrwycr combination that is effective in *in vitro* killing STEC without enhancing release of Shiga toxins.

## Materials and Methods

### Bacterial strains and growth conditions

STEC strain 86–24 (serotype O157:H7; kindly provided by Dr. M. Karmali, Public Health Agency of Canada) and STEC strain, EDL 933 (serotype O157:H7^39^) were used in this study. The STEC 86–24 was isolated during the 1986 Washington state outbreak from a patient who was experiencing hemorrhagic colitis^40^. This strain contains genes encoding only Stx2 and has been associated with the development of severe disease in humans including the development of HUS^41,42^. The EDL933 strain was isolated from a raw hamburger patty that was linked to the 1982 outbreaks in Oregon and Michigan^43^ and carries both *stx*1 and *stx*2 genes^44,45^. It has been noted that STEC strains that express only Stx2 have been more highly associated with the development of HUS than strains that express both Stx1 and Stx2^46–48^. Furthermore, STEC strains that only produced Stx2 were found to be more neurotropic for gnotobiotic piglets than STEC strains that produced only Stx1 or both Stx1 and Stx2^49^. Consequently, STEC 86–24 is generally thought to be more virulent than EDL933. tThus these two strains provide a good comparison for this study.

Bacterial glycerol stocks were maintained at −80 °C. Prior to use, bacteria were streaked for single colonies on Lysogeny Broth (LB) (Bioshop Canada Inc., Burlington, ON, CAN) agar. Single colonies were used to inoculate LB broth for overnight culture at 37 °C with shaking and then subcultured into LB and grown to mid-log phase (OD_600_ of 0.38–0.4) with shaking at 37 °C. Fresh cultures from glycerol stocks were prepared for each experiment in order to maintain the original clinical characteristics of the stock. Bacterial viability was assessed by serial dilution on LB agar.

### Synthesis of wrwycr

The peptide wrwycr was synthesized with a C-terminal amide group, purified to >95% purity at Sigma-Genosys (St. Louis, MO, USA) or Biosynthesis (Lewisville, TX, USA) and dissolved in 50% DMSO as previously described^27^. A wrwycr stock solution (10 mM) was maintained in 50 or 100% DMSO. Final DMSO concentrations were either 0.5% or 1.0% and DMSO at appropriate concentration was added in the absence of wrwycr to control for DMSO effects.

### Minimal inhibitory concentration assay protocol

Prior to the MIC assay, cation-adjusted Mueller Hinton broth (CAMHB; TEKnova, Hollister, CA, USA) media was inoculated with STEC from the overnight LB culture and incubated at 37 °C with 200 rpm shaking to mid-log phase (OD_600_ of 0.3–0.5). Peptide wrwycr and antibiotic concentration ranges were initially selected based on previous reports of MIC values^50^ and then established for this study using the following protocol. Stock solutions (2×) of all antibiotics and peptide solutions are prepared in CAMHB broth and were serially diluted across the microtitre plate, with the last column serving as a ‘no antibiotic’ or ‘full-growth’ control. One column containing no culture served as the ‘no growth’ control. Subcultures in CAMHB media were added to each well to achieve a 5 × 10^5^ cells/mL suspension and incubated statically for 18 hr at 37 °C. The MIC was determined based on the first set of triplicate antibiotic concentrations where no bacterial growth was seen (OD_600_ ≤ 0.01). This protocol is based on CLSI methods for MIC determination.

### Fractional inhibitory concentration index calculations

To establish the MIC values for the peptide wrwycr-antibiotic combinations, a checkerboard assay approach based on CSLI methods was used. In the case of peptide wrwycr-antibiotic combinations, different combinations of peptide wrwycr and antibiotics were prepared using a microdilution 2D checkerboard procedure^51^. The FICI for peptide wrwycr and antibiotic combinations was calculated to determine the nature of inhibitory effects observed in the checkerboard assays. The FICI was calculated for the first non-turbid well (indicating low/no growth) in each row and column of the checkerboard plate, representing the lowest peptide wrwycr/antibiotic combinations that inhibited STEC growth i.e. OD_600_ values ≤ 0.01 were considered non-turbid. The following equation was used: FICI = (MIC_AB_/MIC_A_) + (MIC_AB_/MIC_B_), where MIC_AB_ is the MIC of peptide wrwycr and antibiotic together and MIC_A_ and MIC_B_ are the concentrations of each of the peptide wrwycr and antibiotic alone, respectively. An FICI mean value of less than or equal to 0.5 was considered synergistic while a value greater than 0.5 to less than 4 was considered no interaction/additive and a value greater than or equal to 4 was antagonistic. The FICI mean and range were determined for data at peptide wrwycr and antibiotic concentrations less than their respective MICs where growth was inhibited.

### Time kill assays

To evaluate the effect of the peptide wrwycr and its combination with other antibiotics on bacterial growth, a timed survival/growth curve was constructed according to the standards of the National Committee for Clinical Laboratory Standards (NCCLS). Briefly, an overnight LB culture of STEC was subcultured in CAMHB at 37 °C with shaking to an OD_600_ = 0.38–0.41 (~1 × 10^8^ cells/mL) and then diluted to 5 × 10^5^ cells/mL with CAMHB containing one of peptide wrwycr, antibiotic or peptide wrwycr-antibiotic combination. Samples were incubated with shaking at 37 °C to defined time points (0, 0.5, 1, 2, 3, 5, 7, 12, 24 hr) after which the samples were serially diluted, plated and counted. All assays were carried out in triplicate, and on at least two different occasions.

### Shiga toxin quantification

The effect of treatment of STEC 86–24 with individual antimicrobials or combinations of antimicrobials on Stx production was measured using a Vero cell cytotoxicity assay as described previously^52^. Purified stock solutions of Stx2 (100 mg ml^−1^) were kindly provided by Dr. C. Lingwood, Hospital for Sick Children, Toronto, ON, Canada. Vero cells (ATCC) were cultured at 37 °C, 5% CO_2_ in complete medium (minimal essential medium supplemented with 5–10% fetal bovine serum), seeded at 20,000 cells in 200 mL per well in 96-well plates and cultured overnight to form a monolayer. Serial dilutions of purified Stx2 or Stx extracts from STEC 86–24 samples after various antimicrobial treatments (described below) were prepared in triplicate in complete medium and 50 mL was added per well. The plates were incubated for 72 h after which viable, adherent cells were fixed with 2% formaldehyde in phosphate buffered saline (PBS) and stained with 0.13% crystal violet as described by Gentry & Dalrymple^53^. Crystal violet staining was quantified by absorbance at 595 nm after solubilization of the dye with 50% ethanol in PBS. To prepare the Stx extract, overnight cultures of STEC 86–24 grown in LB broth were subcultured in LB at 37 °C with shaking until mid-exponential phase (OD_600_ = 0.40). 2 ml of LB broth were adjusted with MIC concentrations of either an antibiotics or a peptide wrwycr-antibiotic combination and then inoculated with bacteria (5 × 10^5^ cells/mL) and incubated at 37 °C with shaking. Supernatants (secreted Stx) and periplasmic Stx extracts were taken at two time points, 6 and 15 hr, filter-sterilized and stored at −20 °C. Periplasmic Stx extracts were isolated by resuspending bacterial pellets in 0.5x PBS with polymyxin B (0.1 mg mL^−1^; Sigma Aldrich, Oakville, ON, CAN) and incubating statically at 37 °C for 30 min. *Stx*2 transcription was assessed by quantitative real-time PCR as described previously^17^. Primers RT-*stx2*F (5′-CGACCCCTCTTGAACATA-3′) and RT-*stx2*R (5′-TAGACATCAAGCCCTCGTAT-3′) were used to amplify *stx*2, and primers *gapA*_forward (5′-GTTGTCGCTGAAGCAACTGG-3′) and *gapA*_reverse (5′-AGCGTTGGAAACGATGTCC T-3′) were used to amplify the reference gene *gapA*, encoding d-glyceraldehyde-3-phosphate dehydrogenase A, for normalization.

### Transmission electron microscopy

Five mL of Tryptic Soy Broth (TSB; Millipore Sigma, Oakville, ON, CAN) were inoculated with bacteria and incubated (200 rpm, 37 °C) overnight and then diluted to 0.0025 OD_600_ into 25 mL CAMHB and grown at 37 °C, stationary to OD_600_ of 0.4. Antibiotics/peptide wrwycr/combinations were then added to the bacterial culture and incubated for an additional 2 hr at 37 °C, stationary. Following the incubation, the bacterial pellet was then suspended in 800 μL CAMHB and 200 μL of 2% gluteraldehyde in CAMHB and incubated with rocking overnight at 4 °C. Bacteria were washed 3 times with 0.1 M HEPES buffer, embedded in 2% noble agar and stained for 2 hr in 1% osmium tetroxide. After washing 3 times with 0.1 M HEPES buffer, samples were stained with 1% uranyl acetate (UA; Electron Microscopy Sciences, Hatfield, PA, USA) for 2 hr followed by two washes in 0.1 M HEPES buffer and one wash with double distilled H_2_O (ddH_2_O). Samples were dehydrated by washing with increasing concentrations of ethanol, followed by infiltration into LR white resin. Samples were then solidified into capsules at 60 °C, sectioned and fixed to copper-coated grids. Grids were stained for 10 min with Reynold’s lead citrate (Electron Microscopy Sciences, Hatfield, PA, USA), washed with ddH_2_O, stained with UA for 7 min and finally washed with ddH_2_O and dried before visualizing with a single tilt holder in the FEI Tecnai G2 F20 transmission electron microscope operating at 200 kV and equipped with a bottom-mount Gatan 4k charge-coupled device (CCD) camera.

### Statistical analysis

Graphical presentation and statistical analysis of the data were completed using RStudio version 1.1.453 (RStudio, Inc., Boston, MA, USA). One-way ANOVA and unpaired student’s test were used to differentiate mean values of the time-kill and cytotoxicity assays, respectively. Post hoc comparisons were conducted using Tukey’s test. *P-values* < 0.05 were considered significant.

### Ethical approval and informed consent

There was no work carried out on (i) live vertebrates (or higher invertebrates), (ii) humans or (iii) human samples for this study.

## Supplementary information

Supplementary Information.

## References

[1] Karmali MA, Gannon V, Sargeant JM (2010). Verocytotoxin-producing *Escherichia coli* (VTEC). Veterinary Microbiology.

[2] Melton-Celsa A, Mohawk K, Teel L, O’Brien A (2012). Pathogenesis of Shiga-toxin producing *Escherichia coli*. Curr. Top. Microbiol. Immunol..

[3] Karmali MA (2017). Emerging Public Health Challenges of Shiga Toxin–Producing *Escherichia coli* Related to Changes in the Pathogen, the Population, and the Environment. Clin. Infect. Dis..

[4] Tarr PI, Gordon CA, Chandler WL (2005). Shiga-toxin-producing *Escherichia coli* and hemolytic uremic syndrome. Lancet.

[5] Wong CS (2012). Risk factors for the hemolytic uremic syndrome in children infected with *Escherichia coli* O157:H7: a multivariable analysis. Clin. Infect. Dis..

[6] Palermo MS, Exeni RA, Fernandez GC (2009). Hemolytic uremic syndrome: pathogenesis and update of interventions. Expert Rev. Anti. Infect. Ther..

[7] Karpman, D. & Ståhl, A. Enterohemorrhagic *Escherichia coli* Pathogenesis and the Host Response. In *Enterohemorrhagic Escherichia coli and Other Shiga Toxin-Producing E. coli* vol. 2 403–417 (American Society of Microbiology, 2014). 10.1128/microbiolspec.EHEC-0009-2013 26104359

[8] Withee J (2009). Streamlined analysis for evaluating the use of preharvest interventions intended to prevent *Escherichia coli* O157:H7 illness in humans. Foodborne Pathog. Dis..

[9] Davis TK, McKee R, Schnadower D, Tarr PI (2013). Treatment of shiga toxin-producing *Escherichia coli* infections. Infectious Disease Clinics of North America.

[10] Ardissino G (2016). Early Volume Expansion and Outcomes of Hemolytic Uremic Syndrome. Pediatrics.

[11] Cornfield DN (2016). Shifting the Paradigm in Hemolytic Uremic Syndrome. Pediatrics.

[12] Walterspiel JN, Ashkenazi S, Morrow AL, Cleary TG (1992). Effect of subinhibitory concentrations of antibiotics on extracellular Shiga-like toxin I. Infection.

[13] Yoh, M., Frimpong, E. K. & Honda, T. Effect of antimicrobial agents, especially fosfomycin, on the production and release of verotoxin by enterohemorrhagic *Escherichia coli* O157:H7. *FEMS Immunol. Med. Microbiol.* **19**, 57–64 (1997). 10.1111/j.1574-695X.1997.tb01072.x 9322069

[14] Agger M, Scheutz F, Villumsen S, Mølbak K, Petersen AM (2015). Antibiotic treatment of verocytotoxin-producing *Escherichia coli* (VTEC) infection: a systematic review and a proposal. J. Antimicrob. Chemother..

[15] Bielaszewska M (2012). Effects of antibiotics on Shiga toxin 2 production and bacteriophage induction by epidemic *Escherichia coli* O104:H4 strain. Antimicrob. Agents Chemother..

[16] Zhang Q (2009). Gnotobiotic Piglet Infection Model for Evaluating the Safe Use of Antibiotics against *Escherichia coli* O157:H7 Infection. J. Infect. Dis..

[17] Bielaszewska M (2012). Effects of antibiotics on Shiga toxin 2 production and bacteriophage induction by epidemic *Escherichia coli* O104:H4 strain. Antimicrob. Agents Chemother..

[18] Crane JK, Byrd IW, Boedeker EC (2011). Virulence inhibition by zinc in shiga-toxigenic *Escherichia coli*. Infect. Immun..

[19] Dolgin E (2011). As *E. coli* continues to claim lives, new approaches offer hope. Nat. Med..

[20] Rodrigues DM, Sousa AJ, Johnson-Henry KC, Sherman PM, Gareau MG (2012). Probiotics Are Effective for the Prevention and Treatment of *Citrobacter rodentium*–Induced Colitis in Mice. J. Infect. Dis..

[21] O’Brien AD (1993). Profile of Escherichia coli O157:H7 pathogen responsible for hamburger-borne outbreak of hemorrhagic colitis and hemolytic uremic syndrome in Washington. J. Clin. Microbiol..

[22] O’Brien, A. D. & Melton-Celsa, A. R. New Therapeutic Developments against Shiga Toxin-Producing *Escherichia coli*. *Microbiol. Spectr*. **2** (2014). 10.1128/microbiolspec.EHEC-0013-2013 26104346

[23] Lino, M. *et al*. A novel antimicrobial peptide significantly enhances acid-Induced killing of Shiga toxin-producing *Escherichia coli* O157 and non-O157 serotypes. *Microbiology* **157** (2011). 10.1099/mic.0.047365-0 PMC3167915 21454368

[24] Gunderson CW, Boldt JL, Authement RN, Segall AM (2009). Peptide wrwycr inhibits the excision of several prophages and traps holliday junctions inside bacteria. J. Bacteriol..

[25] Kepple KV, Patel N, Salamon P, Segall AM (2008). Interactions between branched DNAs and peptide inhibitors of DNA repair. Nucleic Acids Res..

[26] Orchard SS, Rostron JE, Segall AM (2012). *Escherichia coli* enterobactin synthesis and uptake mutants are hypersensitive to an antimicrobial peptide that limits the availability of iron in addition to blocking Holliday junction resolution. Microbiology.

[27] Gunderson CW, Segall AM (2006). DNA repair, a novel antibacterial target: Holliday junction-trapping peptides induce DNA damage and chromosome segregation defects. Mol. Microbiol..

[28] Su LY, Willner DL, Segall AM (2010). An antimicrobial peptide that targets DNA repair intermediates *in vitro* inhibits *Salmonella* growth within murine macrophages. Antimicrob. Agents Chemother..

[29] Lackraj T (2016). Novel antimicrobial peptide prevents *C. rodentium infection* in C57BL/6 mice by enhancing acid-induced pathogen killing. Microbiology.

[30] Corogeanu D (2012). Therapeutic concentrations of antibiotics inhibit Shiga toxin release from enterohemorrhagic *E. coli* O104:H4 from the 2011 German outbreak. BMC Microbiol..

[31] Karmali MA (2003). Association of genomic O island 122 of *Escherichia coli* EDL 933 with verocytotoxin-producing *Escherichia coli* seropathotypes that are linked to epidemic and/or serious disease. J. Clin. Microbiol..

[32] Singh R (2005). Identification of antimicrobial resistance and class 1 integrons in Shiga toxin-producing *Escherichia coli* recovered from humans and food animals. J. Antimicrob. Chemother..

[33] Canizalez-Roman A, Gonzalez-Nuñez E, Vidal JE, Flores-Villaseñor H, León-Sicairos N (2013). Prevalence and antibiotic resistance profiles of diarrheagenic *Escherichia coli* strains isolated from food items in northwestern Mexico. Int. J. Food Microbiol..

[34] Skinner C, Zhang G, Patfield S, He X (2015). An *in vitro* combined antibiotic-antibody treatment eliminates toxicity from Shiga toxin-producing *Escherichia coli*. Antimicrob. Agents Chemother..

[35] Wong CS, Jelacic S, Habeeb RL, Watkins SL, Tarr PI (2000). The risk of the hemolytic-uremic syndrome after antibiotic treatment of *Escherichia coli* O157:H7 infections. N. Engl. J. Med..

[36] Goldwater PN, Bettelheim KA (2012). Treatment of enterohemorrhagic *Escherichia coli* (EHEC) infection and hemolytic uremic syndrome (HUS). BMC Med..

[37] Smith KE (2012). Antibiotic Treatment of *Escherichia coli* O157 Infection and the Risk of Hemolytic Uremic Syndrome, Minnesota. Pediatr. Infect. Dis. J..

[38] Corogeanu D (2012). Therapeutic concentrations of antibiotics inhibit Shiga toxin release from enterohemorrhagic *E. coli* O104:H4 from the 2011 German outbreak. BMC Microbiol..

[39] Li Z (1999). Shiga toxin-producing *Escherichia coli* can impair T84 cell structure and function without inducing attaching/effacing lesions. Infect. Immun..

[40] Griffin PM (1988). Illnesses Associated with *Escherichia coli* 0157:H7 Infections: A Broad Clinical Spectrum. Ann. Intern. Med..

[41] Mahan, J. D., Turman, M. A., McCallister, C. & Karmali, M. Verocytotoxin-1 induces apoptosis in human glomerular capillary endothelial cells and mesangial cells *in vitro*. *Pediatr.Res.* **39**, 2168 (1996).

[42] Karmali MA (2003). Association of genomic O island 122 of *Escherichia coli* EDL 933 with verocytotoxin-producing Escherichia coli seropathotypes that are linked to epidemic and/or serious disease. J. Clin. Microbiol..

[43] Wells, J. G. *et al*. Laboratory Investigation of Haemorrhagic Colitis Outbreaks associated with a rare *Escherichia coli* serotype. *J. Clin.Microbiol.* **18**, 512–520 (1983). 10.1128/jcm.18.3.512-520.1983 PMC270845 6355145

[44] Nataro, J. P. & Kaper, J. B. Diarrheagenic. *Escherichia coli. Clin. Microbiol. Rev.* **11**, 141–201 (1998). 10.1128/cmr.11.1.142 PMC121379 9457432

[45] Tzipori, S. *et al*. Role of a 60-Md plasmid and shiga-like toxins in the pathogenesis of infection caused by enterohemorrhagic *Escherichia coli* O157:H7 in gnotobiotic piglets. *Infect. Immun.* **55**, 3117–3125 (1987). 10.1128/iai.55.12.3117-3125.1987 PMC260036 3316033

[46] Boerlin P (1999). Associations between virulence factors of Shiga toxin-producing *Escherichia coli* and disease in humans. J. Clin. Microbiol..

[47] Ethelberg S (2004). Virulence factors for hemolytic uremic syndrome, Denmark. Emerg. Infect. Dis..

[48] Friedrich AW (2002). *Escherichia coli* Harboring Shiga Toxin 2 Gene Variants: Frequency and Association with Clinical Symptoms. J. Infect. Dis..

[49] Donohue‐Rolfe A, Kondova I, Oswald S, Hutto D, Tzipori S (2000). *Escherichia coli* 0157:H7 Strains That Express Shiga Toxin (Stx) 2 Alone Are More Neurotropic for Gnotobiotic Piglets Than Are Isotypes Producing Only Stx1 or Both Stx1 and Stx2. J. Infect. Dis..

[50] Bielaszewska, M., Karmali, M. & Petric, M. Use of the rabbit model to determine the *in vivo* localization of VT2 and to explore the cross reactivity of VT1 and VT2. In *2nd International Symposium and Workshop on Verotoxin-producing E. coli infections* 28 (1994).

[51] Hwang I-s, Hwang JH, Choi H, Kim K-J, Lee DG (2012). Synergistic effects between silver nanoparticles and antibiotics and the mechanisms involved. J. Med. Microbiol..

[52] House B (2009). Acid-stress-induced changes in enterohaemorrhagic *Escherichia coli* O157: H7 virulence. Microbiology.

[53] Gentry MK, Dalrymple JM (1980). Quantitative microtitre cytotoxicity assay for *Shigella* toxin. J. Clin. Microbiol..

