# Supplementary material for: Potentiation of Antibiotics by a Novel Antimicrobial Peptide against Shiga Toxin Producing E. coli O157:H7
Source: Sci Rep. 2020 Jun 22;10:10029. doi: 10.1038/s41598-020-66571-z (PMC7308376; doi:10.1038/s41598-020-66571-z)
Supplement: Supplementary file 1 — Supplementary Information. [file 41598_2020_66571_MOESM1_ESM.pdf]

Supplementary Information:

Potentiation of Antibiotics by a Novel Antimicrobial Peptide against Shiga Toxin Producing *E. coli* O157:H7

Juan Puño-Sarmiento,[a,b#] Erin M. Anderson,[c,d#] Amber J. Park,[c] Cezar M. Khursigara,[c,d] Debora E. Barnett Foster [a,e*]

[a]Department of Chemistry and Biology, Ryerson University, Toronto, Ontario, Canada

[b]Department of Microbiology, Universidade Estadual de Londrina, Londrina, Paraná, Brazil

[c]Department of Molecular and Cellular Biology, University of Guelph, Guelph, Ontario, Canada

[d]Molecular and Cellular Imaging Facility, University of Guelph, Guelph, Ontario, Canada

[e]Oral Microbiology, Faculty of Dentistry, University of Toronto, Toronto, Ontario, Canada

# both authors contributed equally

*Address correspondence to Debora Barnett Foster, dfoster@ryerson.ca

Table S1: Multiple clinical stains are susceptible to combination treatment (P:16 + G:1). Susceptibility assays were conducted for 18 hours and completed for 15 strains, including K12 (nonpathogenic control), 14 clinical STEC isolates from various seropathotypes. Growth vs. K12 values represent OD_{600nm} of untreated cultures of each strain expressed as a % of untreated K12 growth. Values represent OD_{600nm} of untreated or treated cultures of each strain expressed as a % of untreated K12 growth. mean of n=1 biological replicate, n=3 technical replicates.

Figure S1: **Visual Representations of Checkerboard Synergy Assays**. Each panel depicts the level of growth observed (red = no growth, green = growth) for checkerboard assays for combinations of peptide versus (A) gentamicin, (B) kanamycin, (C) chloramphenicol, (D) ciprofloxacin, and (E) meropenem. Yellow stars denote peptide-antibiotic combinations that were further assessed via time-kill assay (as described in Table 3). Numbers within boxes denote calculated FICI values for specific peptide-antibiotic combinations and represent a mean of n=2 biological replicates, n= 3 technical replicates.

Figure S2: Standard Curve of Vero cell Cytotoxicity for Purified Shiga Toxin 2. Cytotoxicity values represent a mean of n=2 biological replicates, n= 3 technical replicates. Data bars represent means ± standard deviation.

Figure S3: Vero Cell Cytotoxicity Levels with Secreted and Periplasmic Lysates from STEC 86-24 following treatment with peptide or antibiotic or peptide-antibiotic combinations after 6 hr and 15 hrs. (A) with subMIC peptide-chloramphenicol combinations with specified FICI values (B) with subMIC peptide-ciprofloxacin combinations with specified FICI values (C) with subMIC

peptide-meropenem combinations with specified FICI values. Cytotoxicity values represent a mean of n=2 biological replicates, n= 3 technical replicates. Data bars represent means ± standard deviation *Significantly different from both individual treatments (peptide or antibiotic); † Significant different from antibiotic treatment. ‡ Significantly different from peptide treatment; $P<0.05$; One-way ANOVA with post hoc Tukey comparisons.

Figure S4: Effect of various treatments on *stx2* gene expression as determined by qRT-PCR. Gene expression was analysed against primers for *gapA* (control) and *stx2*. Results are *stx2* expression relative to normalized reference gene *gapA* and carried out in triplicate. Data bars represent means ± standard deviation.

Figure S5: **Peptide + gentamicin combination reduces cytotoxic response on Vero cells for STEC strain EDL933**. Vero cells were co-cultured for 72 hr with SE collected from untreated bacteria, or bacteria treated for 6 hr with either peptide (64 µg/ml), gentamicin (2 µg/ml), or a combination at P:16 + G:1. Data is mean + standard deviation, n=2-3 biological replicates, n=2 technical replicates per treatment.

Figure S6: STEC strains treated with peptide + gentamicin combination show varying levels of cytotoxicity to Vero cells. Cytotoxicity values are % reduction in dye retention vs. untreated Vero cells (crystal violet, OD_{590nm}). Vero cells were co-cultured for 72hr with SE collected from bacteria treated with a combination of P:16 + G:1. Values represent mean of n=2-3 biological replicates, n=2 technical replicate. Letter under stain IDs indicates seropathotype.

Table S1:

Strain	toxin	seropathotype	growth vs. K12	P:16	G:1	P:16 + G:1
K12	none	n/a	100 ± 0.4	<0.0	<0.0	<0.0
D103F5	stx1,stx2	A	82.4 ± 3.6	<0.0	<0.0	<0.0
158F2	stx1,stx2	B	73.4 ± 2.3	<0.0	<0.0	<0.0
CL1	stx1	B	74.8 ± 3.2	<0.0	<0.0	<0.0
N01-2454	stx1	B	114.4 ± 1.2	0.7 ± 1.2	4.2 ± 6.7	1.0 ± 0.9
CL106	stx1,stx2	B	93.2 ± 5.4	<0.0	<0.0	<0.0
N00-4067	stx1	C	61.6 ± 1.7	1.1 ± 0.7	1.7 ± 0.6	0.1 ± 0.1
G5506	stx2	C	141.9 ± 1.7	2.0 ± 1.8	0.7 ± 0.05	1.8 ± 0.9
CL3	stx2	C	142.0 ± 0.2	16.5 ± 21.1	1.6 ± 0.7	3.8 ± 0.2
N99-4390	stx1,stx2	C	131.3 ± 22.4	25.2 ± 8.9	0.4 ± 0.3	1.8 ± 1.5
EC7-821	stx1	D	92.3 ± 3.9	0.01 ± 0.02	<0.0	<0.0
N02-4495	stx1	D	72.6 ± 2.3	<0.0	<0.0	<0.0
N02-1625	stx1	D	120.5 ± 3.3	17.3 ± 20.2	1.5 ± 0.7	1.4 ± 0.9
A2EV659	stx1,stx2	D	115.9 ± 2.4	22.6 ± 21.3	<0.0	<0.0
EC2-211	stx2	E	69.1 ± 10.9	<0.0	<0.0	<0.0

Figure S1:

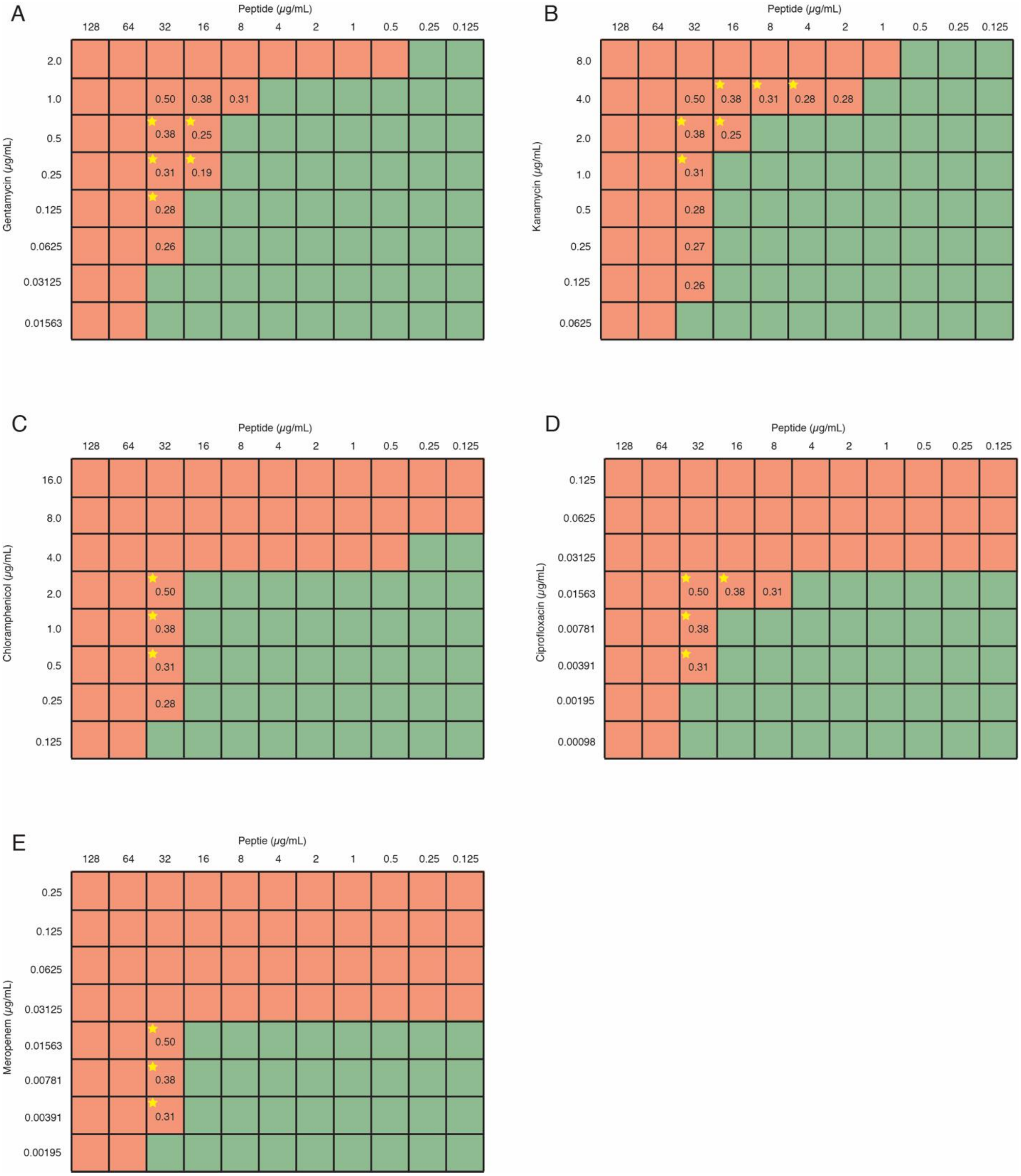
A
Peptide (μg/mL)
128 64 32 16 8 4 2 1 0.5 0.25 0.125
Gentamycin (μg/mL)
2.0 1.0 0.5 0.25 0.125 0.0625 0.03125 0.01563
0.50 0.38 0.31
0.38 0.25
0.31 0.19
0.28
0.26
B
Peptide (μg/mL)
128 64 32 16 8 4 2 1 0.5 0.25 0.125
Kanamycin (μg/mL)
8.0 4.0 2.0 1.0 0.5 0.25 0.125 0.0625
0.50 0.38 0.31 0.28 0.28
0.38 0.25
0.31
0.28
0.27
0.26
C
Peptide (μg/mL)
128 64 32 16 8 4 2 1 0.5 0.25 0.125
Chloramphenicol (μg/mL)
16.0 8.0 4.0 2.0 1.0 0.5 0.25 0.125
0.50
0.38
0.31
0.28
D
Peptide (μg/mL)
128 64 32 16 8 4 2 1 0.5 0.25 0.125
Ciprofloxacin (μg/mL)
0.125 0.0625 0.03125 0.01563 0.00781 0.00391 0.00195 0.00098
0.50 0.38 0.31
0.38
0.31
E
Peptie (μg/mL)
128 64 32 16 8 4 2 1 0.5 0.25 0.125
Meropenem (μg/mL)
0.25 0.125 0.0625 0.03125 0.01563 0.00781 0.00391 0.00195
0.50
0.38
0.31

Figure S2:

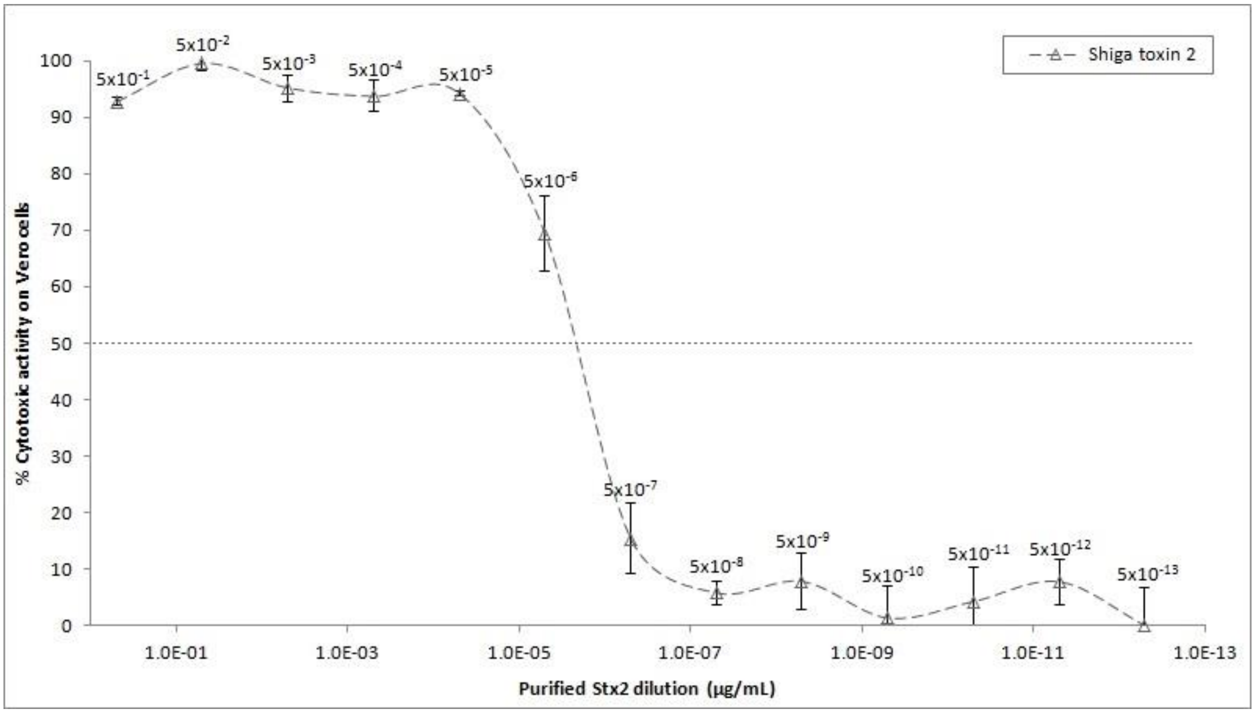

Shiga toxin 2
% Cytotoxic activity on Vero cells
Purified Stx2 dilution (μg/mL)
0
10
20
30
40
50
60
70
80
90
100
1.0E-01
1.0E-03
1.0E-05
1.0E-07
1.0E-09
1.0E-11
1.0E-13
5x10-1
5x10-2
5x10-3
5x10-4
5x10-5
5x10-6
5x10-7
5x10-8
5x10-9
5x10-10
5x10-11
5x10-12
5x10-13

Figure S3:

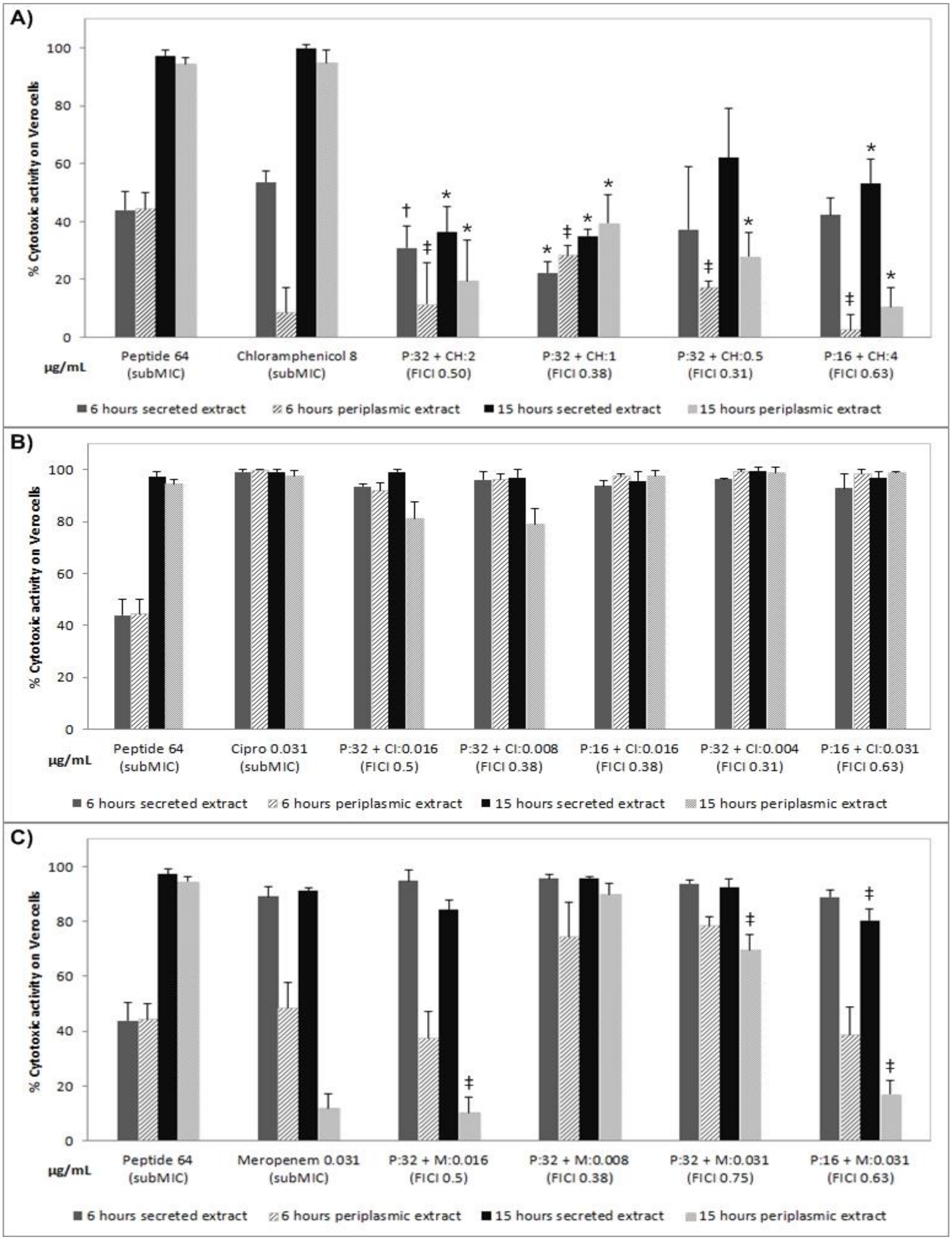
A)
% Cytotoxic activity on Vero cells
µg/mL
Peptide 64 (subMIC)
Chloramphenicol 8 (subMIC)
P:32 + CH:2 (FICI 0.50)
P:32 + CH:1 (FICI 0.38)
P:32 + CH:0.5 (FICI 0.31)
P:16 + CH:4 (FICI 0.63)
6 hours secreted extract
6 hours periplasmic extract
15 hours secreted extract
15 hours periplasmic extract
B)
% Cytotoxic activity on Vero cells
µg/mL
Peptide 64 (subMIC)
Cipro 0.031 (subMIC)
P:32 + CI:0.016 (FICI 0.5)
P:32 + CI:0.008 (FICI 0.38)
P:16 + CI:0.016 (FICI 0.38)
P:32 + CI:0.004 (FICI 0.31)
P:16 + CI:0.031 (FICI 0.63)
6 hours secreted extract
6 hours periplasmic extract
15 hours secreted extract
15 hours periplasmic extract
C)
% Cytotoxic activity on Vero cells
µg/mL
Peptide 64 (subMIC)
Meropenem 0.031 (subMIC)
P:32 + M:0.016 (FICI 0.5)
P:32 + M:0.008 (FICI 0.38)
P:32 + M:0.031 (FICI 0.75)
P:16 + M:0.031 (FICI 0.63)
6 hours secreted extract
6 hours periplasmic extract
15 hours secreted extract
15 hours periplasmic extract

Figure S4:

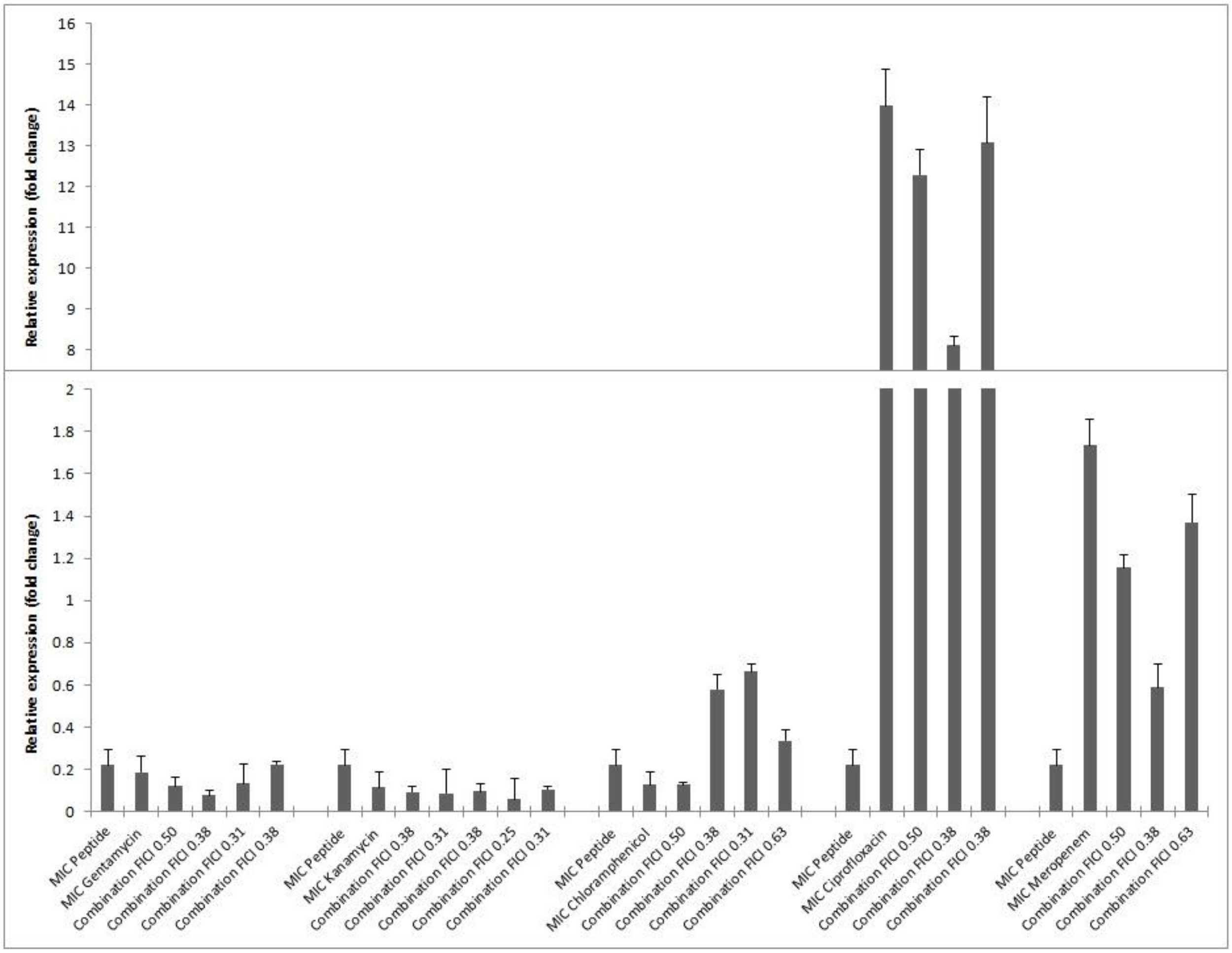

Relative expression (fold change)
16
15
14
13
12
11
10
9
8
Relative expression (fold change)
2
1.8
1.6
1.4
1.2
1
0.8
0.6
0.4
0.2
0
MIC Peptide
MIC Gentamycin
Combination FICI 0.50
Combination FICI 0.38
Combination FICI 0.31
Combination FICI 0.38
MIC Peptide
MIC Kanamycin
Combination FICI 0.38
Combination FICI 0.31
Combination FICI 0.38
Combination FICI 0.25
Combination FICI 0.31
MIC Peptide
MIC Chloramphenicol
Combination FICI 0.50
Combination FICI 0.38
Combination FICI 0.31
Combination FICI 0.63
MIC Peptide
MIC Ciprofloxacin
Combination FICI 0.50
Combination FICI 0.38
Combination FICI 0.38
MIC Peptide
MIC Meropenem
Combination FICI 0.50
Combination FICI 0.38
Combination FICI 0.63

Figure S5

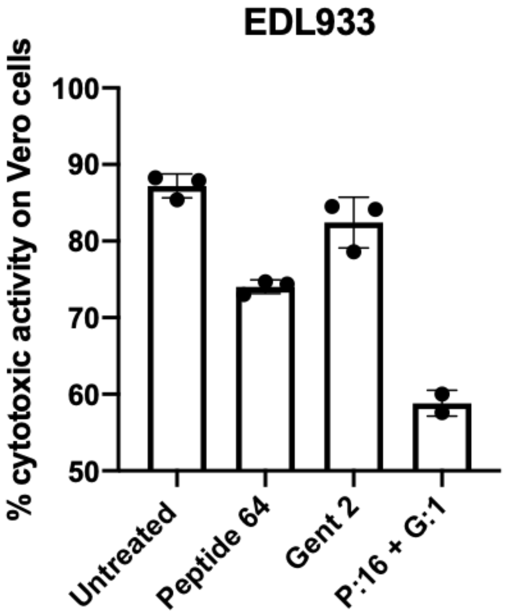
EDL933
% cytotoxic activity on Vero cells
100
90
80
70
60
50
Untreated
Peptide 64
Gent 2
P:16 + G:1

Figure S6

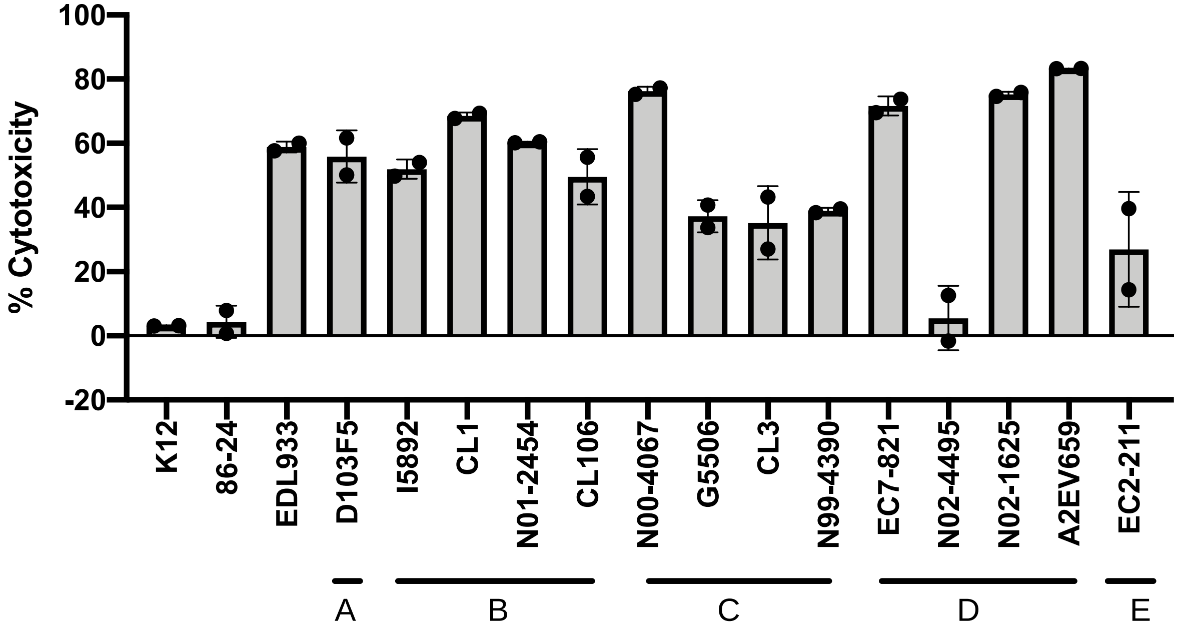

% Cytotoxicity
100
80
60
40
20
0
-20
K12
86-24
EDL933
D103F5
I5892
CL1
N01-2454
CL106
N00-4067
G5506
CL3
N99-4390
EC7-821
N02-4495
N02-1625
A2EV659
EC2-211
A
B
C
D
E
